# Hydrogen-bonded salt cocrystals of xenon difluoride and protonated perfluoroamides†

**DOI:** 10.1039/d5ce00956a

**Published:** 2025-11-04

**Authors:** Erik Uran, Matic Lozinšek

**Affiliations:** a Extreme Condition Chemistry Laboratory (ECCL K2), Jožef Stefan Institute Jamova cesta 39 1000 Ljubljana Slovenia matic.lozinsek@ijs.si; b Jožef Stefan International Postgraduate School Jamova cesta 39 1000 Ljubljana Slovenia

## Abstract

The hydrogen-bonding ability of XeF_2_ is an important factor influencing its chemical properties and reactivity, yet structurally characterised examples of hydrogen-bonded xenon fluorides remain rare. In this work, three salt cocrystals containing hydrogen-bonded xenon difluoride and hexafluoridoarsenate salts of protonated perfluoroamides—CF_3_C(OH)NH_2_[AsF_6_]·XeF_2_, C_2_F_5_C(OH)NH_2_[AsF_6_]·XeF_2_, and C_3_F_7_C(OH)NH_2_[AsF_6_]·XeF_2_—were synthesised and structurally characterised. Diverse hydrogen-bonding motifs were observed, and the first crystallographically characterised examples of N–H⋯FXeF hydrogen bonds are presented. In total, eleven new crystal structures are reported, including two perfluoroamides, three protonated and two hemiprotonated perfluoroamides, and one salt cocrystal containing an oxonium ion. The XeF_2_-containing cocrystals demonstrate that XeF_2_ reliably functions as a hydrogen-bond acceptor and readily forms hydrogen-bonded cocrystals. These findings broaden the scope of noble-gas chemistry and highlight the potential of noble-gas fluorides for cocrystal formation.

## Abstract 2

The hydrogen-bonding ability of XeF_2_ is an important factor influencing its chemical properties and reactivity, yet structurally characterised examples of hydrogen-bonded xenon fluorides remain rare.

## Graphical abstract

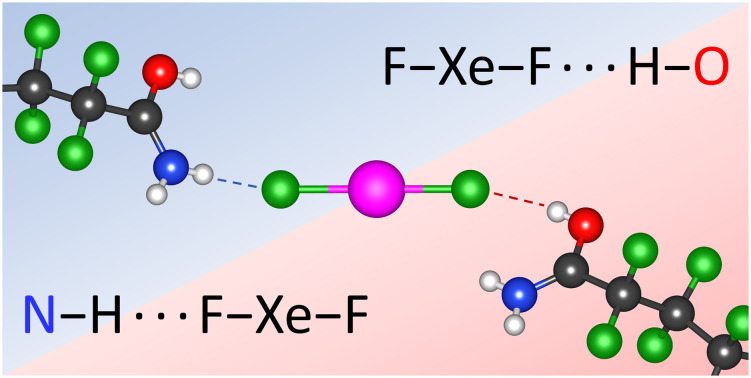

## Introduction

Xenon difluoride (XeF_2_) is the most common and extensively studied binary noble-gas fluoride and serves as a precursor to a wide range of xenon compounds.^1,2^ It is a nonpolar molecular compound with linear geometry. XeF_2_ is a good fluoride-ion donor^3^ and thus forms a variety of Lewis acid–base adducts^4^ and a plethora of coordination compounds with metal cations.^1,2,5^

The ability of XeF_2_ to act as a hydrogen-bond acceptor strongly influences its physical and chemical properties. As a nonpolar molecule, it is highly soluble in the polar protic solvent anhydrous HF (aHF) (167 g/100 g at 30 °C).^6^ This unusually high solubility arises from the formation of FXe–F⋯HF hydrogen bonds.^7–9^ Furthermore, XeF_2_ dissolved in aHF is a considerably more potent oxidiser than pure XeF_2_, and even trace amounts of HF can catalyse its reactions with organic substrates through the hydrogen-bonding induced polarisation [FXe^*δ*+^–F^*δ*−^⋯HF].^10^ HF also facilitates fluorine exchange in XeF_2_, enabling the synthesis of ^18^F-radiolabelled XeF_2_.^11,12^ In certain cases, the influence of HF is so pronounced that it can unexpectedly alter reaction outcomes, even when inadvertently generated by reaction with the vessel material.^13^

Despite the ability of XeF_2_ to act as a hydrogen-bond acceptor, systematic crystallographic investigations are absent, and reported solid-state examples remain scarce. To date, only a handful of crystallographically characterised examples of hydrogen-bonded XeF_2_ have been described. These include O–H⋯FXeF hydrogen bonds observed in H_3_O[AsF_6_]·2XeF_2_ and in HNO_3_·XeF_2_ cocrystals,^14,15^ as well as an F–H⋯FXeF interaction observed in the coordination complex [Cd(HF)_2_(XeF_2_)(MF_6_)_2_] (M = Ta, Nb).^5,16^

It has also been shown spectroscopically that protonated trifluoroacetamide (CF_3_CONH_2_) forms a hydrogen-bonded salt cocrystal^17^ with XeF_2_, CF_3_C(OH)NH_2_[AsF_6_]·XeF_2_·*x*HF.^18^ This cocrystal is particularly noteworthy, as it may feature both =OH^+^ and –NH_2_ groups as hydrogen-bond donors,^18^ potentially offering insight into the hydrogen-bonding preferences of XeF_2_.

To investigate the hydrogen-bonding propensity of XeF_2_ in the solid state and its tendency to form cocrystals with NH and OH hydrogen-bond donors, the crystal structures of XeF_2_ salt cocrystals with protonated CF_3_CONH_2_, C_2_F_5_CONH_2_, and C_3_F_7_CONH_2_ were studied in this work. The perfluoroamides were selected because of their anticipated resistance to oxidative-fluorination by XeF_2_.

## Results and discussion

### Crystal structures of CF_3_CF_2_CONH_2_ and CF_3_CF_2_CF_2_CONH_2_

The crystal structures of pentafluoropropionamide (C_2_F_5_CONH_2_) and heptafluorobutyramide (C_3_F_7_CONH_2_) were elucidated by low-temperature single-crystal X-ray diffraction (LT SCXRD) (Tables 1 and S1), whereas the crystal structure of CF_3_CONH_2_ has been previously reported at 295 K and 110 K.^19,20^ For comparison of bond lengths (Table S2), only the structure obtained at 110 K was considered.^20^

**Table 1:** Summary of crystal data and refinement results for crystal structures of amides and protonated amides

Compound	C_2_F_5_CONH_2_	C_3_F_7_CONH_2_	CF_3_C(OH)NH_2_[AsF_6_]	C_2_F_5_C(OH)NH_2_[AsF_6_]	C_3_F_7_C(OH)NH_2_[AsF_6_]
Space group	*C*2/*c*	*P*1̄	*P*2_1_/*c*	*Pccn*	*P*2_1_/*c*
*a* (Å)	21.7871(5)	5.11713(18)	9.81910(18)	8.12957(13)	6.17592(15)
*b* (Å)	5.11704(12)	5.27137(14)	7.90095(13)	25.2768(4)	7.94187(19)
*c* (Å)	10.0754(3)	12.7768(3)	20.5015(4)	9.34322(16)	21.7914(5)
*α* (°)	90	95.467(2)	90	90	90
*β* (°)	98.140(2)	91.890(3)	98.6498(18)	90	96.014(2)
*γ* (°)	90	105.584(3)	90	90	90
*V* (Å^3^)	1111.94(5)	329.847(18)	1572.42(5)	1919.93(5)	1062.95(4)
*M*	163.06	213.07	302.97	352.98	402.99
*Z*	8	2	8	8	4
*T* (K)	100	100	100	100	100
*R*[*F*^2^ > 2*σ*(*F*^2^)]	0.048	0.028	0.033	0.027	0.027
w*R*(*F*^2^)	0.138	0.076	0.087	0.058	0.068

**Fig. 1:**
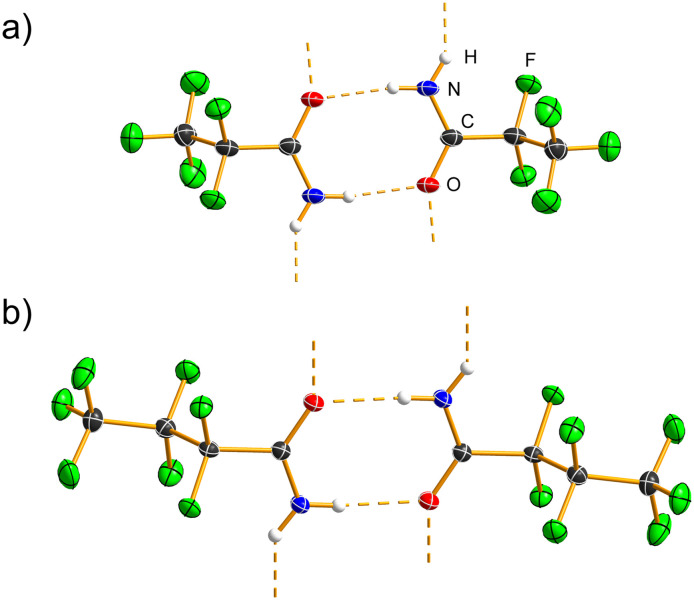
R^2^_2_(8) hydrogen-bonding motifs in the crystal structures of (a) C_2_F_5_CONH_2_ and (b) C_3_F_7_CONH_2_. Hydrogen bonds are shown as dashed orange lines. Displacement ellipsoids are drawn at the 50% probability level, and hydrogen atoms are represented as spheres of arbitrary radius.

C_2_F_5_CONH_2_ (Fig. 1a and S1) crystallizes in the monoclinic space group *C*2/*c* with *Z* = 8. The C=O bond length (1.2323(19) Å) is comparable to the distances observed in the crystal structures of other primary amides, and the same applies to the C–N bond (1.317(2) Å).^21^ Two N–H⋯O hydrogen bonds (2.912(2) Å, 171(2)°; 2.8396(17) Å, 149(2)°; Table S3) in the crystal structure form R^2^_2_(8) and R^4^_6_(16) hydrogen-bonding motifs,^22^ which assemble into a corrugated layer parallel to the *bc* plane (Fig. S2 and S3).

C_3_F_7_CONH_2_ (Fig. 1b and S4) crystallizes in the triclinic space group *P*1̄ with *Z* = 2. The C=O bond distance (1.2293(15) Å) is essentially identical to that in C_2_F_5_CONH_2_, as is the C–N bond (1.3162(16) Å). These bond distances are shorter than the corresponding ones observed in non-fluorinated secondary amides, such as capsaicin.^23^ Two N–H⋯O hydrogen bonds (Table S4) are present in the crystal structure (2.9313(14) Å, 174.3(16)°; 2.8495(14) Å, 140.4(15)°), which fall within the typical range for amide molecules.^21^ The R^2^_2_(8) and R^2^_4_(8) hydrogen-bond motifs link the molecules into a ladder along the *a*-crystallographic axis (Fig. S5).

### Protonation of amides in superacidic media HF–AsF_5_

All amides are soluble in aHF and readily undergo protonation upon addition of AsF_5_. In all cases, protonation occurs at the oxygen atom, consistent with previous observations.^18,24–26^ Low-temperature crystallisation from aHF afforded crystals of suitable quality for SCXRD.

**Fig. 2:**
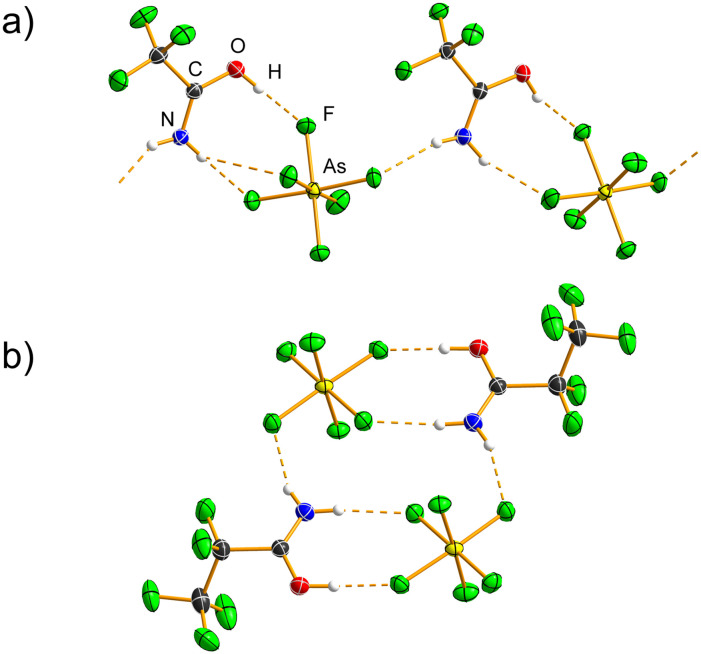
Crystal structure of (a) the hydrogen-bonded chain in CF_3_C(OH)NH_2_[AsF_6_] and (b) the discrete hydrogen-bonded cluster in C_2_F_5_C(OH)NH_2_[AsF_6_] (only one orientation of the disordered –C_2_F_5_ unit is shown). Hydrogen bonds are shown as dashed orange lines. Displacement ellipsoids are drawn at the 50% probability level, and hydrogen atoms are represented as spheres of arbitrary radius.

CF_3_C(OH)NH_2_[AsF_6_] (Tables 1, S1 and S2; Fig. S6) crystallises in the monoclinic space group *P*2_1_/*c* with *Z* = 8 and *Z*′ = 2. Upon protonation, the C=O bonds (1.2795(19), 1.282(2) Å) lengthen and the C–N bonds (1.279(2), 1.281(2) Å) shorten relative to those in CF_3_CONH_2_ (1.2304(12) and 1.3164(13) Å, respectively).^20^ These changes in the C=O and C–N bond lengths are consistent with previous crystallographic studies of protonated amides.^25–27^ The O–H⋯F hydrogen bonds (2.5860(16) Å, 2.6530(18) Å; Table S5) bracket the value observed in CF_3_C(OH)NH_2_[SbF_6_] (2.600(1) Å), whereas the N–H⋯F hydrogen bonds (2.8236(18)–3.0797(18) Å) are comparable to those in CF_3_C(OH)NH_2_[SbF_6_] (2.884(2), 2.933(2) Å).^26^ All hydrogen-bond angles (121(2)–179(3)°) fall within the typical range. The [AsF_6_]^−^ anions deviate from ideal octahedral geometry, with the longest As–F bonds (1.7524(10), 1.7557(10) Å) participating in hydrogen bonding with =OH^+^ group. In the crystal structure, cations and anions are linked through O–H⋯F and N–H⋯F hydrogen-bonded chains (Fig. 2a and S7).

C_2_F_5_C(OH)NH_2_[AsF_6_] (Tables 1, S1 and S2) crystallises in the orthorhombic space group *Pccn* with *Z* = 8 and features a disordered –C_2_F_5_ moiety (Fig. S8). Perfluorinated alkyl chains frequently exhibit disorder in the crystalline state,^28^ as F⋯F interactions are relatively weak,^29^ and can therefore adopt various conformations. The C=O (1.2821(15) Å) and C–N (1.2772(16) Å) bonds are longer and shorter, respectively, than those in C_2_F_5_CONH_2_. The [AsF_6_]^−^ anion deviates from ideal octahedral geometry, with the *mer*-As–F bonds involved in hydrogen bonding being longer (1.7253(8)–1.7453(8) Å) than the remaining As–F bonds (1.6976(8)–1.7111(8) Å). The hydrogen bonds (Table S6) between the =OH^+^ and –NH_2_ groups and the [AsF_6_]^−^ anions (O(H)⋯F, 2.6006(12) Å, 172(2)°; N(H)⋯F, 2.8309(13) Å, 174(2)° and 2.8316(14) Å, 161.3(19)°) lead to the formation of discrete units (Fig. 2b, S8 and S9), exhibiting R^2^_2_(8) and R^4^_4_(12) hydrogen-bonding motifs.

**Fig. 3:**
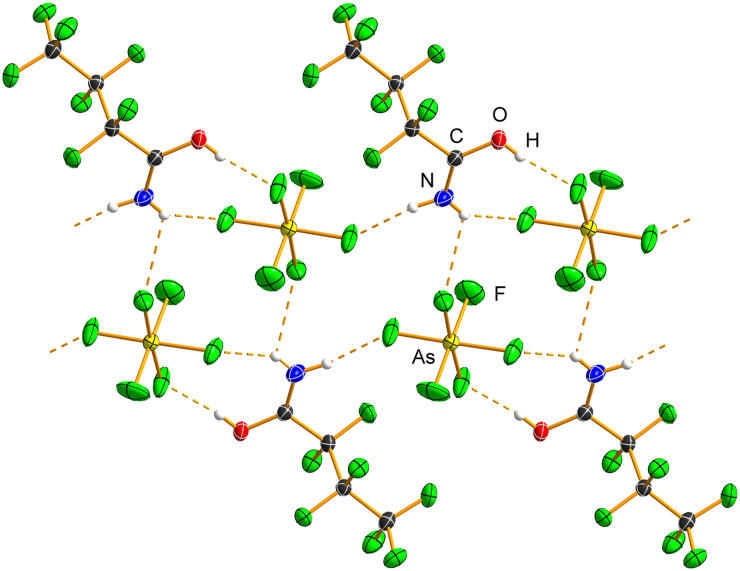
Hydrogen-bonded ribbon in C_3_F_7_C(OH)NH_2_[AsF_6_] (only one orientation of the disordered [AsF_6_]^−^ anion is shown). Hydrogen bonds are shown as dashed orange lines. Displacement ellipsoids are drawn at the 50% probability level, and hydrogen atoms are represented as spheres of arbitrary radius.

C_3_F_7_C(OH)NH_2_[AsF_6_] (Tables 1, S1 and S2) crystallises in the monoclinic space group *P*2_1_/*c* with *Z* = 4, with the [AsF_6_]^−^ anion disordered over two positions (Fig. S10). The C=O bond (1.2797(14) Å) is elongated, and the C–N bond (1.2841(16) Å) is shortened compared to those in C_3_F_7_CONH_2_. A similar C=O(H) bond distance (1.274(2) Å) was observed in the crystal structure of (C_6_F_5_)_2_COH[AsF_6_].^30^ Hydrogen bonds (Table S7) are formed between the =OH^+^ group (2.541(3), 2.557(3) Å; 157(3), 165(3)°) or the –NH_2_ group (2.737(4)–3.179(5) Å, 118.0(19)–168.1(19)°) and the [AsF_6_]^−^ anions. The O⋯F hydrogen bond is the shortest among the protonated amides in this study, and also shorter than those in CH_3_C(OH)NH_2_[AsF_6_]^25^ and CF_3_C(OH)NH_2_[SbF_6_].^26^ The [AsF_6_]^−^ anion deviates from ideal octahedral geometry (1.642(3)–1.795(2) Å). The C_3_F_7_C(OH)NH_2_^+^ cations and [AsF_6_]^−^ anions are linked into a hydrogen-bonded ribbon (Fig. 3 and S11), exhibiting conjoined R^4^_4_(12), R^4^_2_(8) and R^2^_2_(8) motifs.

**Fig. 4:**
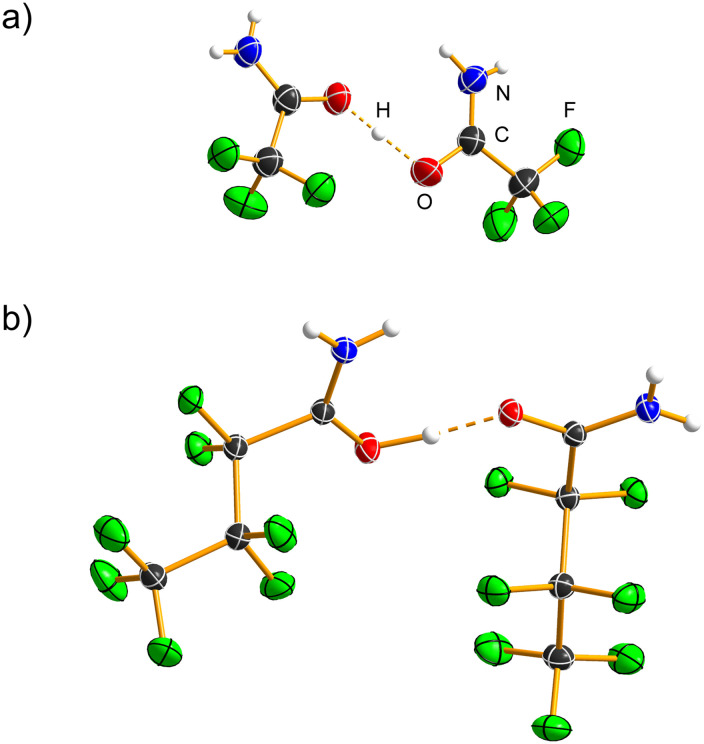
Hydrogen-bonded dimers in the crystal structure of (a) (CF_3_CONH_2_)_2_H[AsF_6_] and (b) (C_3_F_7_CONH_2_)_2_H[AsF_6_]. The short O–H⋯O=C hydrogen bonds are shown as dashed orange lines. Displacement ellipsoids are drawn at the 50% probability level, and hydrogen atoms are represented as spheres of arbitrary radius.

Two hemiprotonated salts, (CF_3_CONH_2_)_2_H[AsF_6_] and (C_3_F_7_CONH_2_)_2_H[AsF_6_] (Fig. 4 and S12–S17; Tables 2, S1 and S2), were also crystallographically characterised. The former was inadvertently found during the low-temperature crystal selection and mounting of the CF_3_C(OH)NH_2_[AsF_6_]·XeF_2_ sample, whereas the latter was identified as an impurity in the sample of C_3_F_7_C(OH)NH_2_[AsF_6_]·XeF_2_ salt cocrystals. Both compounds crystallise in the triclinic space group *P*1̄ with *Z* = 2. In both structures, the C=O bonds are elongated (1.283(6) Å in (CF_3_CONH_2_)_2_H[AsF_6_]; 1.2652(9), 1.2459(9) Å in (C_3_F_7_CONH_2_)_2_H[AsF_6_]), whereas the C–N bonds are shortened (1.274(7) Å in (CF_3_CONH_2_)_2_H[AsF_6_]; 1.2942(10), 1.3027(10) Å in (C_3_F_7_CONH_2_)_2_H[AsF_6_]) compared to the non-protonated amides.^20^ The values for one of the amide molecules in (CF_3_CONH_2_)_2_H[AsF_6_] fall within the range for neutral amide,^20^ owing to the relatively high standard uncertainties of the bond lengths. The O(H)⋯O hydrogen bond length in (CF_3_CONH_2_)_2_H[AsF_6_] (2.426(5) Å, 170(8)°) is essentially identical to that in (C_3_F_7_CONH_2_)_2_H[AsF_6_] (2.4174(9) Å, 172(2)°) (Tables S8 and S9), and comparable to literature values for such hydrogen-bonded systems.^21^ The nearly equidistant position of the hydrogen atom (O–H, H⋯O: 1.13(9), 1.31(9) Å in (CF_3_CONH_2_)_2_H[AsF_6_]; 1.06(2), 1.36(2) Å in (C_3_F_7_CONH_2_)_2_H[AsF_6_]), together with the relatively short O⋯O distances, indicates strong, positive charge-assisted hydrogen bonding, (+)CAHB.^31^ These structures represent rare examples of proton sharing between two primary amide molecules,^21,32^ a motif more commonly observed in secondary and tertiary amides.^21^ The –NH_2_ groups are hydrogen-bonded to [AsF_6_]^−^ anions (N⋯F, 2.644(5)–3.005(5) Å in (CF_3_CONH_2_)_2_H[AsF_6_]; 2.8168(9)–3.1046(9) Å in (C_3_F_7_CONH_2_)_2_H[AsF_6_]) (Fig. S13–S17), resulting in the formation of ribbons that are further interconnected by the anions into layers parallel to the *ab* plane.

**Table 2:** Summary of crystal data and refinement results for hemiprotonated amides and H_3_O[AsF_6_]·2CF_3_CONH_2_ salt cocrystal

Compound	(CF_3_CONH_2_)_2_H[AsF_6_]	(C_3_F_7_CONH_2_)_2_H[AsF_6_]	H_3_O[AsF_6_]·2CF_3_CONH_2_
Space group	*P*1̄	*P*1̄	*Pnma*
*a* (Å)	5.2815(3)	5.32051(4)	11.5349(2)
*b* (Å)	10.1517(6)	10.45222(9)	14.0649(3)
*c* (Å)	12.4911(6)	16.10170(12)	7.78994(14)
*α* (°)	108.936(5)	90.5740(6)	90
*β* (°)	93.107(5)	90.8760(6)	90
*γ* (°)	102.904(5)	103.0402(7)	90
*V* (Å^3^)	611.63(6)	872.156(12)	1263.82(4)
*M*	416.02	616.06	434.04
*Z*	2	2	4
*T* (K)	100	100	100
*R*[*F*^2^ > 2*σ*(*F*^2^)]	0.047	0.022	0.037
w*R*(*F*^2^)	0.125	0.055	0.085

**Fig. 5:**
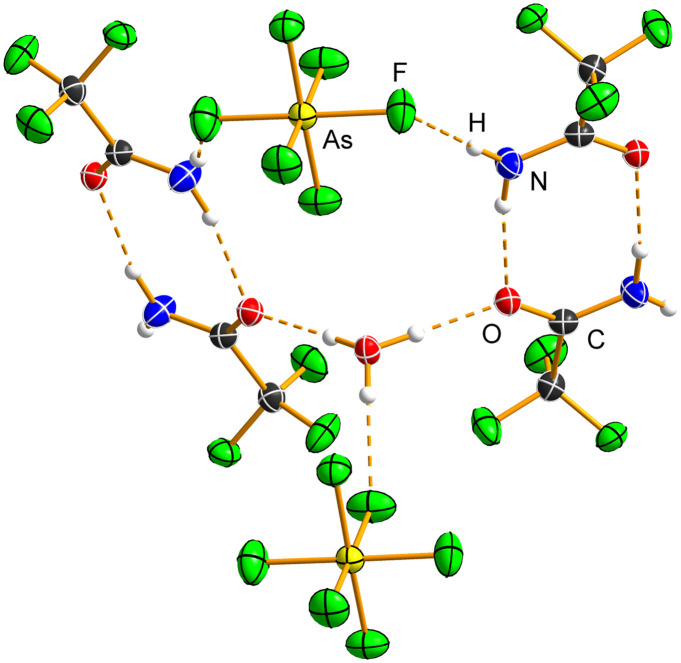
R^4^_6_(14) hydrogen-bonded cluster in the crystal structure of the H_3_O[AsF_6_]·2CF_3_CONH_2_ salt cocrystal. Hydrogen bonds are shown by dashed orange lines. Displacement ellipsoids are drawn at the 50% probability level, and hydrogen atoms are represented as spheres of arbitrary radius.

A crystal of H_3_O[AsF_6_]·2CF_3_CONH_2_ (Tables 2, S1 and S2; Fig. 5 and S18–S20) was fortuitously found during the low-temperature crystal selection and mounting of the CF_3_C(OH)NH_2_[AsF_6_]·XeF_2_ sample. It crystallises in the orthorhombic space group *Pnma* with *Z* = 4. The amide molecule is not protonated, resulting in C=O (1.236(4) Å) and C–N (1.304(4) Å) bond lengths that are close to those in CF_3_CONH_2_.^20^ The amide molecule acts as both a hydrogen-bond donor and acceptor (Table S10, Fig. S19), forming N–H⋯F(As) and N–H⋯O(C) hydrogen bonds. An R^2^_2_(8) motif is observed between two amide molecules, with the N⋯O hydrogen bond (2.959(4) Å, 171(4)°) comparable to that found in CF_3_CONH_2_.^20^ The H_3_O^+^ cation forms three hydrogen bonds: two symmetrically equivalent O–H⋯O(C) (2.525(3) Å, 167(4)°) and one O–H⋯F hydrogen bond (2.657(5) Å, 173(7)°) with the [AsF_6_]^−^ anion. Together, these hydrogen bonds form a hydrogen-bonded cluster represented by R^6^_6_(20), R^4^_6_(14) and R^2^_2_(8) graph-set motifs^22^ (Fig. 5), which further extend into a layer parallel to the *bc* plane (Fig. S20).

### Hydrogen-bonded salt cocrystals of XeF_2_

The reaction of amides with equimolar amounts of [XeF][AsF_6_] at temperatures down to −30 °C leads to the formation of RC(OH)NH_2_[AsF_6_]·XeF_2_ salt cocrystals. This indicates that a proton from HF is transferred to the amide, generating a protonated amide, while the resulting fluoride anion reacts with [XeF]^+^ to form XeF_2_. This behaviour was also reported in a previous study of the CF_3_CONH_2_–[XeF][AsF_6_] system.^18^

The salt cocrystals (Tables 3, S1 and S2) thus feature protonated amides cocrystallised with XeF_2_ and exhibit a rare O–H⋯FXeF hydrogen bond, as well as the first crystallographically characterised examples of N–H⋯FXeF hydrogen bonds.

**Table 3:** Summary of crystal data and refinement results for salt cocrystals of protonated amides with XeF_2_

Compound	CF_3_C(OH)NH_2_[AsF_6_]·XeF_2_	C_2_F_5_C(OH)NH_2_[AsF_6_]·XeF_2_	C_3_F_7_C(OH)NH_2_[AsF_6_]·XeF_2_
Space group	*P*2_1_/*n*	*Aea*2	*Pnna*
*a* (Å)	7.41785(9)	8.67561(10)	8.62011(14)
*b* (Å)	9.84875(11)	31.0125(4)	35.5418(5)
*c* (Å)	14.90113(17)	8.65174(9)	8.71910(12)
*α* (°)	90	90	90
*β* (°)	99.4517(11)	90	90
*γ* (°)	90	90	90
*V* (Å^3^)	1073.85(2)	2327.77(4)	2671.31(7)
*M*	472.27	522.28	572.29
*Z*	4	8	8
*T* (K)	100	100	100
*R*[*F*^2^ > 2*σ*(*F*^2^)]	0.025	0.018	0.026
w*R*(*F*^2^)	0.067	0.038	0.066

**Fig. 6:**
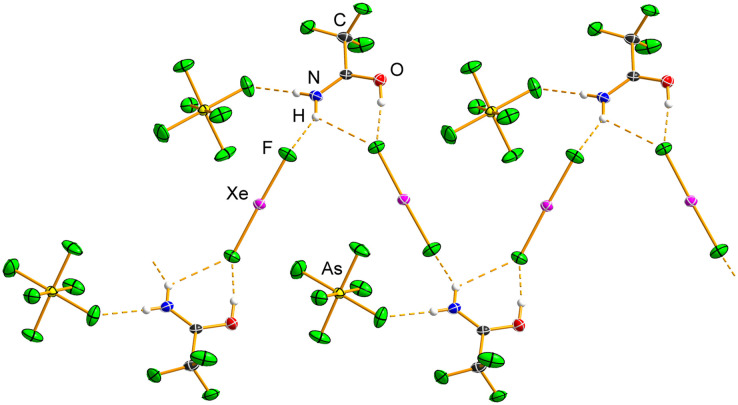
Hydrogen-bonded ribbon in the crystal structure of the salt cocrystal CF_3_C(OH)NH_2_[AsF_6_]·XeF_2_. Only one orientation of the disordered –CF_3_ moiety is shown. Hydrogen bonds are shown as dashed orange lines. Displacement ellipsoids are drawn at the 50% probability level, and hydrogen atoms are represented as spheres of arbitrary radius.

CF_3_C(OH)NH_2_[AsF_6_]·XeF_2_ (Fig. 6 and S21) crystallises in the monoclinic space group *P*2_1_/*n* with *Z* = 4. The XeF_2_ molecule exhibits slight asymmetry in Xe–F bond distances (1.9669(10), 2.0237(9) Å) compared to pure XeF_2_ (1.999(4) Å),^33^ and it remains linear (178.10(5) Å). The asymmetry of XeF_2_ is slightly smaller than that observed in XeF_2_·HNO_3_ (1.9737(8), 2.0506(8) Å).^15^

CF_3_C(OH)NH_2_[AsF_6_]·XeF_2_ is the only salt cocrystal in this series that exhibits both O–H⋯F(Xe) and N–H⋯F(Xe) hydrogen bonds. One fluorine atom of XeF_2_ is acting as a bifurcated acceptor (Fig. 6 and S21; Table S11). The O–H⋯F(Xe) hydrogen bond (2.5467(14) Å, 171(3)°) is shorter than that in H_3_O[AsF_6_]·2XeF_2_ (2.571(3) Å)^14^ and HNO_3_·XeF_2_ (2.690(1) Å).^15^ It is also significantly shorter than the O–H⋯F(As) hydrogen bonds in CF_3_C(OH)NH_2_[AsF_6_] and C_2_F_5_C(OH)NH_2_[AsF_6_], but comparable to that in C_3_F_7_C(OH)NH_2_[AsF_6_]. The N–H⋯F(Xe) hydrogen bonds (2.7865(15) Å, 151(3)°; 3.0894(16), 124(2)°), which involve a single bifurcated donor, are longer than those observed in the other two salt cocrystals described in this study. The C=O (1.2773(15) Å) and C–N (1.2772(16) Å) bond lengths are essentially identical to those in the protonated salts,^26^ indicating a negligible influence of hydrogen bonding on the overall geometry of the CF_3_C(OH)NH_2_^+^ cation. The –CF_3_ moiety is disordered, as also observed in the crystal structure of CF_3_CONH_2_.^20^ The [AsF_6_]^−^ anion participates in hydrogen bonding with the –NH_2_ group (2.8270(18) Å, 176(3)°; 3.0594(15) Å, 111(2)°), resulting in a slight deviation from ideal octahedral geometry (As–F, 1.7006(12)–1.7417(11) Å).

Hydrogen bonds between CF_3_C(OH)NH_2_^+^ and XeF_2_ form a zigzag chain parallel to the *b*-crystallographic axis, with pendant [AsF_6_]^−^ anions connected to the chain *via* N–H⋯F(As) hydrogen bonds, giving rise to a ribbon-like structure (Fig. 6 and S22).

Both C_2_F_5_C(OH)NH_2_[AsF_6_]·XeF_2_ and C_3_F_7_C(OH)NH_2_[AsF_6_]·XeF_2_ (Tables 3, S1 and S2; Fig. S23–S26) crystallise in orthorhombic space groups, *Aea*2 and *Pnna*, respectively, with *Z* = 8. The asymmetry of the Xe–F bond lengths in C_2_F_5_C(OH)NH_2_[AsF_6_]·XeF_2_ (1.9734(14), 2.0061(15) Å) and in C_3_F_7_C(OH)NH_2_[AsF_6_]·XeF_2_ (1.9674(15), 2.0135(16) Å) is comparable. The shorter Xe–F bonds are similar to that observed in the trifluoroacetamide analogue, whereas the longer Xe–F bonds are significantly shorter. In both cocrystals, the F–Xe–F angle is essentially linear (179.88(9)°; 179.57(7)°).

The N–H⋯F(Xe) hydrogen bonds (Tables S12 and S13) in C_2_F_5_C(OH)NH_2_[AsF_6_]·XeF_2_ (2.688(3), 2.729(3) Å) and C_3_F_7_C(OH)NH_2_[AsF_6_]·XeF_2_ (2.692(3), 2.737(3) Å) are comparable and are significantly shorter than the corresponding hydrogen bonds in CF_3_C(OH)NH_2_[AsF_6_]·XeF_2_. The C=O (1.289(2), 1.285(3) Å) and C–N (1.279(3), 1.280(3) Å) bond lengths in C_2_F_5_C(OH)NH_2_[AsF_6_]·XeF_2_ and C_3_F_7_C(OH)NH_2_[AsF_6_]·XeF_2_ are almost identical to those observed in the corresponding protonated salts.

**Fig. 7:**
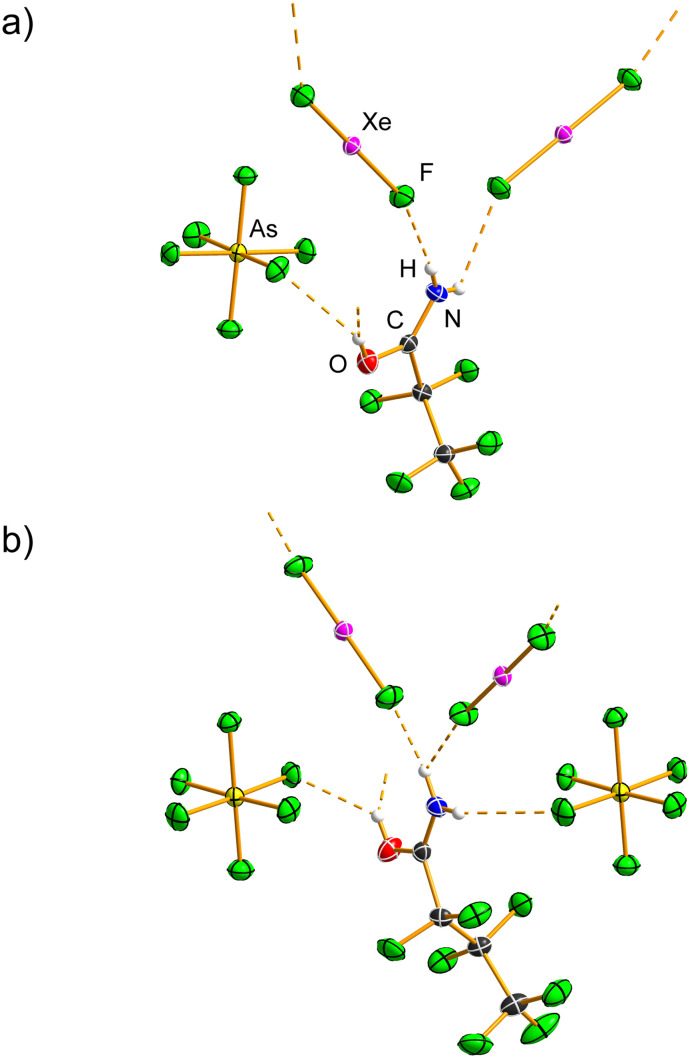
Hydrogen bonds (dashed orange lines) in the crystal structures of the salt cocrystals (a) C_2_F_5_C(OH)NH_2_[AsF_6_]·XeF_2_ and (b) C_3_F_7_C(OH)NH_2_[AsF_6_]·XeF_2_. The OH groups are bifurcated hydrogen-bond donors; however, two [AsF_6_]^−^ anions have been omitted for clarity. Displacement ellipsoids are drawn at the 50% probability level, and hydrogen atoms are represented as spheres of arbitrary radius.

The protonated oxygen atom acts as a hydrogen-bond donor towards the [AsF_6_]^−^ anions, forming bifurcated hydrogen bonds (Fig. 7, S23 and S25), which are longer than the O–H⋯F(As) hydrogen bonds observed in the parent protonated salts.

The packing in both C_2_F_5_C(OH)NH_2_[AsF_6_]·XeF_2_ and C_3_F_7_C(OH)NH_2_[AsF_6_]·XeF_2_ consists of hydrogen-bonded ribbons composed of alternating protonated amide and XeF_2_ molecules, similar to those observed in CF_3_C(OH)NH_2_[AsF_6_]·XeF_2_. These ribbons are further connected by O–H⋯F(As) hydrogen bonds, and in the case of C_3_F_7_C(OH)NH_2_[AsF_6_]·XeF_2_, also by N–H⋯F(As) hydrogen bonds (Fig. S24 and S26).

The relatively small difference in Xe–F bond lengths in the present XeF_2_ cocrystals suggests that hydrogen bonding has only a minor influence on XeF_2_ ionisation (XeF_2_ → XeF^+^ + F^−^).^34^ In particular, the shorter Xe–F bonds (1.9669(10)–1.9734(14) Å) are considerably longer than those found in [XeF]^+^ tight ion pairs^33,35,36^ and in [Xe_2_F_3_]^+^ salts.^35,37^ They are comparable to the shortest Xe–F bond lengths in XeF_2_ adduct-salts with [BrOF_2_]^+^ (1.956(5), 1.960(4) Å)^38^ and [BrO_2_]^+^ cations (1.970(4)–1.978(3) Å).^39^ Nevertheless, the distortion of hydrogen-bonded XeF_2_ observed in the present salt cocrystals is significant when compared with Xe–F bond distances observed in the crystal structures containing cocrystallised XeF_2_, *e.g.*, 3XeF_2_·2MnF_4_ (1.9933(7) Å),^36^ and in the molecular cocrystals XeF_2_·XeF_4_ (1.9940(9) Å)^37^ and XeF_2_·XeOF_4_ (2.014(5) Å),^40^ in which XeF_2_ is centrosymmetric.

### Vibrational spectroscopy

**Fig. 8:**
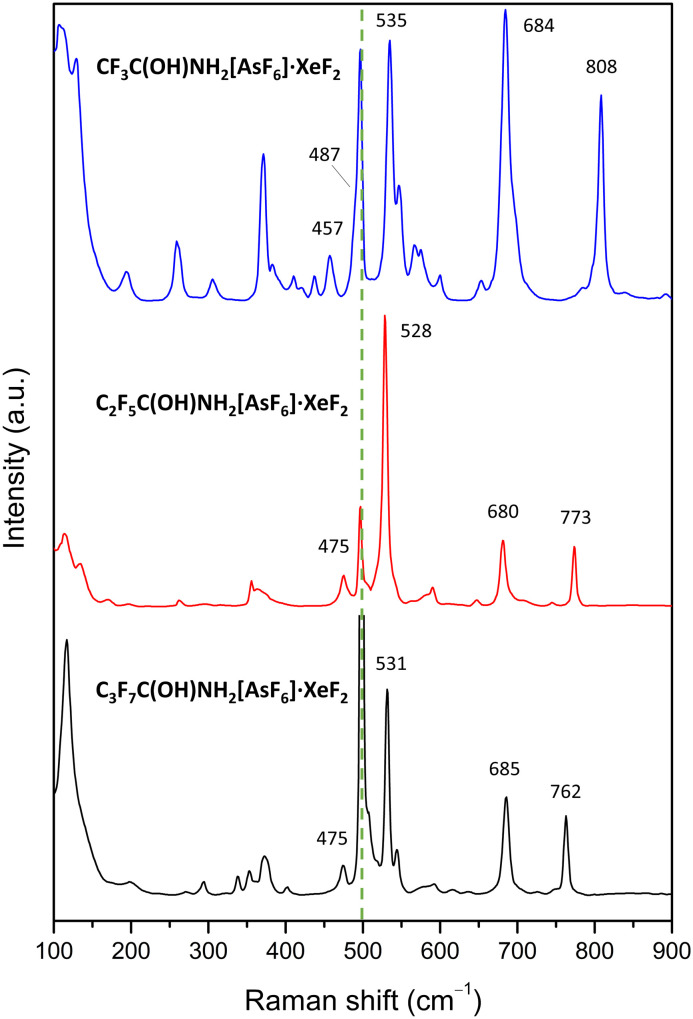
Raman spectra of XeF_2_ salt cocrystals with protonated amides recorded at low temperatures (−90 °C). The green dashed line is placed at the position of free XeF_2_ (497 cm^−1^)^41^ which was observed as an impurity in the reactions.

To corroborate the findings from LT SCXRD and to gain further insight into the ionisation of XeF_2_, low-temperature Raman spectra were measured (Fig. 8 and S27–S40). Two bands at 457–475 and 528–535 cm^−1^ are observed in all XeF_2_ salt cocrystals in this study, corresponding to the elongated and shortened Xe–F bond, respectively. These bands are significantly shifted from that of pure XeF_2_ (497 cm^−1^)^41^ and from values observed when cocrystallised XeF_2_ does not participate in significant intermolecular interactions, such as in XeF_2_·XeOF_4_ (494, 503 cm^−1^),^40^ XeF_2_·XeF_4_ (505 cm^−1^),^42^ 3XeF_2_·2MnF_4_ (508 cm^−1^),^36^ and XeF_2_·N_2_O_4_ (509 cm^−1^).^15^ The value of the higher-frequency band is comparable to the Raman shifts reported for the adduct salts [BrOF_2_][AsF_6_]·XeF_2_ (531, 543, 559 cm^−1^),^38^ [BrO_2_][AsF_6_]·*n*XeF_2_ (*n* = 1, 2; 516–546 cm^−1^),^39^ and for the hydrogen-bonded cocrystals H_3_O[AsF_6_]·2XeF_2_ (552 cm^−1^)^14^ and HNO_3_·XeF_2_ (529 cm^−1^).^15^ However, these shifts are significantly smaller than those observed in [XeF]^+^ tight-ion pair salts (>600 cm^−1^) and [Xe_2_F_3_]^+^ cations (580–600 cm^−1^).^1,33,35,36,43,44^ The band around 535 cm^−1^ is particularly noteworthy, as this value coincides with that observed for XeF_2_ dissolved in aHF, which has been attributed to the FXe–F⋯HF hydrogen bonds.^7–9^

In addition to the bands attributed to XeF_2_, those arising from [AsF_6_]^−^ anions are observed around 375 and 680 cm^−1^.^18,25,26,45^ Vibrations from the protonated amide molecules are also present, including an intense band around 800 cm^−1^ corresponding to ν(C–C),^25,26^ and peaks near 1100 cm^−1^ attributed to C–F vibrations.^18,26,46^ In all protonated amides, the N–H stretching vibrations were observed in 3150–3400 cm^−1^ range.^18,26,46^

## Experimental

**Caution!** Anhydrous HF, AsF_5_, XeF_2_, [XeF][AsF_6_] and the compounds prepared in this study are highly reactive and hazardous. The amides used may cause skin, eye, and respiratory irritation. Contact with the skin must be avoided, and all compounds should be handled exclusively in a well-ventilated fume hood.

Appropriate safety precautions must be observed at all times, and working with minimal quantities is strongly recommended.

## Materials and methods

Reactions were carried out in fluorinated ethylene propylene (FEP) vessels equipped with Kel-F or PTFE valves. All vessels were passivated with fluorine prior to use. Volatile substances were handled using a fluorine-resistant metal vacuum line, whereas solids were manipulated inside an N_2_-filled glovebox. Detailed synthetic procedures are provided in the SI. Characterisation was performed by low-temperature single-crystal X-ray diffraction and low-temperature Raman spectroscopy. Single-crystal selection and mounting were carried out using a low-temperature crystal-mounting apparatus, as described previously (SI).^30,36,47^ Low-temperature Raman spectra were recorded directly on the aluminium trough used for mounting single crystals for X-ray diffraction measurements.

## Conclusions

In this work, the perfluoroamides trifluoroacetamide (CF_3_CONH_2_), pentafluoropropionamide (C_2_F_5_CONH_2_), and heptafluorobutyramide (C_3_F_7_CONH_2_), were protonated in superacidic medium HF–AsF_5_, and the crystal structures of the resulting salts, CF_3_C(OH)NH_2_[AsF_6_], C_2_F_5_C(OH)NH_2_[AsF_6_], and C_3_F_7_C(OH)NH_2_[AsF_6_] were elucidated. Protonation at the carbonyl oxygen atom is consistently observed. In addition, the crystal structures of the amides C_2_F_5_CONH_2_ and C_3_F_7_CONH_2_, the hemiprotonated salts (CF_3_CONH_2_)_2_H[AsF_6_] and (C_3_F_7_CONH_2_)_2_H[AsF_6_], and the oxonium salt cocrystal H_3_O[AsF_6_]·2CF_3_CONH_2_ were determined. Low-temperature reactions of the perfluoroamides with [XeF][AsF_6_] in aHF yielded rare XeF_2_-containing salt cocrystals: CF_3_C(OH)NH_2_[AsF_6_]·XeF_2_, C_2_F_5_C(OH)NH_2_[AsF_6_]·XeF_2_ and C_3_F_7_C(OH)NH_2_[AsF_6_]·XeF_2_. Their crystal structures reveal a rare example of O–H⋯FXeF and the first crystallographically characterised cases of N–H⋯FXeF hydrogen bonding. The XeF_2_ molecule is slightly polarised, as indicated by the differences observed in Xe–F bond lengths compared with those in free XeF_2_; this finding is corroborated by low-temperature Raman spectroscopy. The reported crystal structures display diverse hydrogen-bonding motifs involving O–H⋯F(Xe), N–H⋯F(Xe), O–H⋯F(As) and N–H⋯F(As) interactions. The salt cocrystals prepared and structurally characterised in this study demonstrate that XeF_2_ readily forms hydrogen-bonded cocrystals and serves as a reliable hydrogen-bond acceptor. These results open new possibilities for the exploration of cocrystal formation with noble-gas fluorides and the expansion of noble-gas chemistry.

## Author contributions

Conceptualization, data curation, formal analysis, investigation, visualization, writing – original draft: EU; funding acquisition, methodology, project administration, resources, supervision: ML; validation, writing – review & editing: EU, ML. Both authors agreed on the final version of the article.

## Conflicts of interest

There are no conflicts to declare.

## Supplementary Material

CE-027-D5CE00956A-s001

CE-027-D5CE00956A-s002

## Data Availability

Supplementary information: crystallographic details, Raman spectra, experimental details. See DOI: https://doi.org/10.1039/d5ce00956a. Crystallographic data for all reported crystal structures has been deposited at the Cambridge Crystallographic Data Centre (CCDC) under deposition numbers 2493130–2493140.^48*a–k*^ Data for this article, including SCXRD datasets and Raman spectra are available at Zenodo open repository at https://doi.org/10.5281/zenodo.17432981.
