# Supplementary material for: Hydrogen-bonded salt cocrystals of xenon difluoride and protonated perfluoroamides
Source: CrystEngComm. 2025 Nov 4;27(48):7776–84. doi: 10.1039/d5ce00956a (PMC12659783; doi:10.1039/d5ce00956a)
Supplement: CE-027-D5CE00956A-s001 [file CE-027-D5CE00956A-s001.pdf]

Supplementary Information (SI) for CrystEngComm.
This journal is © The Royal Society of Chemistry 2025

Supplementary Information

# Hydrogen-Bonded Salt Cocrystals of Xenon Difluoride and Protonated Perfluoroamides

Erik Uran,[a,b] Matic Lozinšek*[a,b]

[a] *Extreme Condition Chemistry Laboratory (ECCL K2), Jožef Stefan Institute, Jamova cesta 39, 1000 Ljubljana, Slovenia*

[b] *Jožef Stefan International Postgraduate School, Jamova cesta 39, 1000 Ljubljana, Slovenia*

* Corresponding author: matic.lozinsek@ijs.si

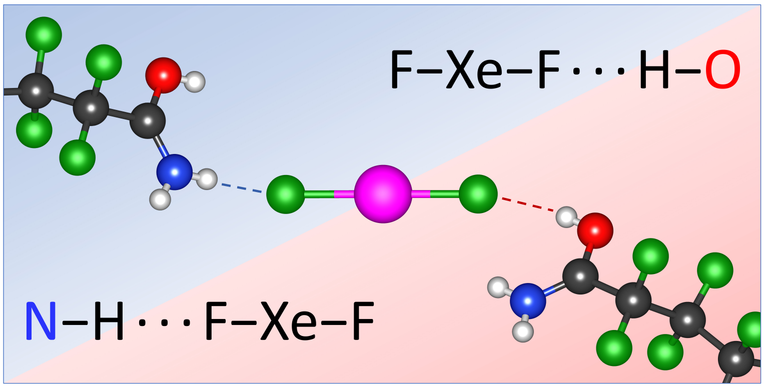


DOI: 10.1039/D5CE00956A

# Table of Contents


**1. Crystallographic details** ........ 4
**Table S1.** Summary of crystal data and structure refinements ........ 4
**Table S2**. C=O, C−N, (O)C−C bond lengths and N−C−O angles . ........ 8
**Figure S1**. Asymmetric unit of the $C_2F_5CONH_2$ crystal structure ........ 9
**Figure S2**. Hydrogen bonds in $C_2F_5CONH_2$ ........ 9
**Table S3**. Hydrogen-bond geometry in $C_2F_5CONH_2$ ........ 9
**Figure S3**. Hydrogen-bonded corrugated layer in the crystal structure of $C_2F_5CONH_2$ ........ 9
**Figure S4**. Asymmetric unit of the $C_3F_7CONH_2$ crystal structure ........ 10
**Figure S5**. Hydrogen bonds, packing diagram and unit cell of $C_3F_7CONH_2$ ........ 10
**Table S4**. Hydrogen-bond geometry in $C_3F_7CONH_2$ ........ 10
**Figure S6**. Expanded asymmetric unit of $CF_3C(OH)NH_2[AsF_6]$ and hydrogen bonds. ........ 11
**Table S5**. Hydrogen-bond geometry in $CF_3C(OH)NH_2[AsF_6]$ ........ 11
**Figure S7**. Packing diagram and the unit cell of the $CF_3C(OH)NH_2[AsF_6]$ ........ 11
**Figure S8**. Expanded asymmetric unit of $C_2F_5C(OH)NH_2[AsF_6]$ and hydrogen bonds ........ 12
**Table S6**. Hydrogen-bond geometry in $C_2F_5C(OH)NH_2[AsF_6]$ ........ 12
**Figure S9**. Packing diagram and unit cell of $C_2F_5C(OH)NH_2[AsF_6]$ ........ 12
**Figure S10**. Expanded asymmetric unit of $C_3F_7C(OH)NH_2[AsF_6]$ and hydrogen bonds ........ 13
**Table S7**. Hydrogen-bond geometry in $C_3F_7C(OH)NH_2[AsF_6]$ ........ 13
**Figure S11**. Packing diagram and unit cell of $C_3F_7C(OH)NH_2[AsF_6]$ ........ 13
**Figure S12**. Asymmetric unit of $(CF_3CONH_2)_2H[AsF_6]$. ........ 14
**Figure S13**. Hydrogen bonds in $(CF_3CONH_2)_2H[AsF_6]$. ........ 14
**Table S8**. Hydrogen-bond geometry in $(CF_3CONH_2)_2H[AsF_6]$ ........ 14
**Figure S14**. Packing diagram and unit cell of $(CF_3CONH_2)_2H[AsF_6]$ ........ 14
**Figure S15**. Asymmetric unit of $(C_3F_7CONH_2)_2H[AsF_6]$ ........ 15
**Figure S16**. Hydrogen bonds in $(C_3F_7CONH_2)_2H[AsF_6]$ . ........ 15
**Table S9**. Hydrogen-bond geometry in $(C_3F_7CONH_2)_2H[AsF_6]$ ........ 15
**Figure S17**. Packing diagram and unit cell of $(C_3F_7CONH_2)_2H[AsF_6]$ ........ 15
**Figure S18**. Asymmetric unit of $H_3O[AsF_6]\cdot 2CF_3C(O)NH_2$ ........ 16
**Figure S19**. Hydrogen bonds in $H_3O[AsF_6]\cdot 2CF_3CONH_2$ ........ 16
**Table S10**. Hydrogen bond geometry in $H_3O[AsF_6]\cdot 2CF_3CONH_2$ ........ 16
**Figure S20**. Packing diagram and unit cell of $H_3O[AsF_6]\cdot 2CF_3CONH_2$ ........ 16
**Figure S21**. Expanded asymmetric unit of $CF_3C(OH)NH_2[AsF_6]\cdot XeF_2$ and hydrogen bonds ........ 17
**Table S11**. Hydrogen-bond geometry in $CF_3C(OH)NH_2[AsF_6]\cdot XeF_2$ ........ 17
**Figure S22**. Packing diagram and unit cell of $CF_3C(OH)NH_2[AsF_6]\cdot XeF_2$ ........ 17
**Figure S23**. Expanded asymmetric unit of $C_2F_5C(OH)NH_2[AsF_6]\cdot XeF_2$ and hydrogen bonds. ........ 18
**Table S12.** Hydrogen-bond geometry in $C_2F_5C(OH)NH_2[AsF_6]\cdot XeF_2$ ........ 18
**Figure S24**. Packing diagram and the unit cell of $C_2F_5C(OH)NH_2[AsF_6]\cdot XeF_2$ ........ 18
**Figure S25**. Expanded asymmetric unit of $C_3F_7C(OH)NH_2[AsF_6]\cdot XeF_2$ and hydrogen bonds. ........ 19
**Table S13.** Hydrogen-bond geometry in $C_3F_7C(OH)NH_2[AsF_6]\cdot XeF_2$ ........ 19

**2. Low-temperature Raman spectra** ........ 20
**Figure S27**. Raman spectrum of $CF_3CONH_2$ (−90 °C) ........ 20
**Figure S28**. Raman spectrum of $CF_3C(OH)NH_2[AsF_6]$ (−100 °C) ........ 21
**Figure S29**. Raman spectrum of $CF_3C(OH)NH_2[AsF_6]\cdot XeF_2$ (−90 °C) ........ 22

**Figure S30**. Comparison of Raman spectra of $CF_3CONH_2$, $CF_3C(OH)NH_2[AsF_6]$, and $CF_3C(OH)NH_2[AsF_6]\cdot XeF_2$. ... 23
**Figure S31**. Raman spectrum of $C_2F_5CONH_2$ (−90 °C) ... 24
**Figure S32**. Raman spectrum of $C_2F_5C(OH)NH_2[AsF_6]$ (−50 °C) ... 25
**Figure S33**. Raman spectrum of $C_2F_5C(OH)NH_2[AsF_6]\cdot XeF_2$ (−90 °C) ... 26
**Figure S34**. Raman spectra of $C_2F_5CONH_2$, $C_2F_5C(OH)NH_2[AsF_6]$, and $C_2F_5C(OH)NH_2[AsF_6]\cdot XeF_2$ ... 27
**Figure S35**. Raman spectrum of $C_3F_7CONH_2$ (−90 °C) ... 28
**Figure S36**. Raman spectrum of $C_3F_7C(OH)NH_2[AsF_6]$ (−100 °C) ... 29
**Figure S37**. Raman spectrum of a mixture of $(C_3F_7CONH_2)_2H[AsF_6]$ and $C_3F_7C(OH)NH_2[AsF_6]\cdot XeF_2$ ... 30
**Figure S38**. Enlarged Raman spectrum from Figure S37 ... 31
**Figure S39**. Comparison of Raman spectra of $C_3F_7CONH_2$, $C_3F_7C(OH)NH_2[AsF_6]$, and a mixture of $C_3F_7C(OH)NH_2[AsF_6]\cdot XeF_2$ and $(C_3F_7CONH_2)_2H[AsF_6]$ ... 32
**Figure S40**. Comparison of Raman spectra of $CF_3C(OH)NH_2[AsF_6]\cdot XeF_2$, $C_2F_5C(OH)NH_2[AsF_6]\cdot XeF_2$, and $C_3F_7C(OH)NH_2[AsF_6]\cdot XeF_2$ ... 33

3. Experimental details ... 34
**Figure S41**. Experimental and calculated powder diffractogram of $CF_3C(OH)NH_2[AsF_6]\cdot XeF_2$ ... 36

## 1. Crystallographic details

**Table S1.** Summary of crystal data and structure refinements

| Compound | $C_2F_5CONH_2$ | $C_3F_7CONH_2$ |
|---|---|---|
| Chemical formula | $C_3H_2F_5NO$ | $C_4H_2F_7NO$ |
| $M_r$ | 163.06 | 213.07 |
| Crystal system | Monoclinic | Triclinic |
| Space group | *C*2/*c* | *P*−1 |
| $T$ / K | 100 | 100 |
| $a$ / Å | 21.7871 (5) | 5.11713 (18) |
| $b$ / Å | 5.11704 (12) | 5.27137 (14) |
| $c$ / Å | 10.0754 (3) | 12.7768 (3) |
| α / ° | 90 | 95.467 (2) |
| β / ° | 98.140 (2) | 91.890 (3) |
| γ / ° | 90 | 105.584 (3) |
| $V$ / Å$^3$ | 1111.94 (5) | 329.847 (18) |
| $Z$ | 8 | 2 |
| Radiation type | Cu Kα | Cu Kα |
| μ / mm$^{-1}$ | 2.33 | 2.62 |
| Crystal size / mm | 0.17 × 0.12 × 0.06 | 0.68 × 0.12 × 0.05 |
| $T_{min}$, $T_{max}$ | 0.651, 1.000 | 0.425, 1.000 |
| No. of measured, independent and observed [$I > 2\sigma(I)$] reflections | 15587, 1161, 1063 | 6558, 1342, 1292 |
| $R_{int}$ | 0.047 | 0.023 |
| $\sin(\theta/\lambda)_{max}$ / Å$^{-1}$ | 0.630 | 0.629 |
| $R[F^2 > 2\sigma(F^2)]$, $wR(F^2)$, $S$ | 0.048, 0.138, 1.12 | 0.028, 0.076, 1.06 |
| No. of reflections | 1161 | 1342 |
| No. of parameters | 99 | 127 |
| No. of restraints | / | / |
| H-atom treatment | All H-atom parameters refined | All H-atom parameters refined |
| $\Delta\rho_{max}$, $\Delta\rho_{min}$ / e Å$^{-3}$ | 0.39, −0.35 | 0.35, −0.22 |

**Table S1.** Continued …

| Compound | $CF_3C(OH)NH_2[AsF_6]$ | $C_2F_5C(OH)NH_2[AsF_6]$ | $C_3F_7C(OH)NH_2[AsF_6]$ |
|---|---|---|---|
| Chemical formula | $C_2H_3F_9NOAs$ | $C_3H_3AsF_{11}NO$ | $C_4H_3AsF_{13}NO$ |
| $M_r$ | 302.97 | 352.98 | 402.99 |
| Crystal system | Monoclinic | Orthorhombic | Monoclinic |
| Space group | $P2_1/c$ | *Pccn* | $P2_1/c$ |
| $T$ / K | 100 | 100 | 100 |
| $a$ / Å | 9.81910 (18) | 8.12957 (13) | 6.17592 (15) |
| $b$ / Å | 7.90095 (13) | 25.2768 (4) | 7.94187 (19) |
| $c$ / Å | 20.5015 (4) | 9.34322 (16) | 21.7914 (5) |
| α / ° | 90 | 90 | 90 |
| β / ° | 98.6498 (18) | 90 | 96.014 (2) |
| γ / ° | 90 | 90 | 90 |
| $V$ / Å$^3$ | 1572.42 (5) | 1919.93 (5) | 1062.95 (4) |
| $Z$ | 8 | 8 | 4 |
| Radiation type | Ag Kα, λ = 0.56087 Å | Ag Kα, λ = 0.56087 Å | Ag Kα, λ = 0.56087 Å |
| μ / mm$^{-1}$ | 2.35 | 1.95 | 1.79 |
| Crystal size / mm | 0.11 × 0.08 × 0.06 | 0.58 × 0.4 × 0.17 | 0.64 × 0.35 × 0.25 |
| $T_{min}$, $T_{max}$ | 0.845, 1.000 | 0.233, 1.000 | 0.277, 1.000 |
| No. of measured, independent and observed [$I > 2\sigma(I)$] reflections | 80745, 8411, 6219 | 89392, 5471, 4374 | 48154, 5105, 4403 |
| $R_{int}$ | 0.050 | 0.043 | 0.047 |
| $\sin(\theta/\lambda)_{max}$ / Å$^{-1}$ | 0.893 | 0.907 | 0.833 |
| $R[F^2 > 2\sigma(F^2)]$, $wR(F^2)$, $S$ | 0.033, 0.087, 1.03 | 0.027, 0.058, 1.09 | 0.027, 0.068, 1.05 |
| No. of reflections | 8411 | 5471 | 5105 |
| No. of parameters | 277 | 221 | 249 |
| No. of restraints | / | 28 | / |
| H-atom treatment | All H-atom parameters refined | All H-atom parameters refined | All H-atom parameters refined |
| $\Delta\rho_{max}$, $\Delta\rho_{min}$ / e Å$^{-3}$ | 0.88, −0.72 | 0.60, −0.52 | 0.57, −0.84 |

**Table S1.** Continued …

| Compound | $(CF_3CONH_2)_2H[AsF_6]$ | $(C_3F_7CONH_2)_2H[AsF_6]$ | $H_3O[AsF_6]\cdot 2CF_3CONH_2$ |
|---|---|---|---|
| Chemical formula | $C_4H_5AsF_{12}N_2O_2$ | $C_8H_5AsF_{20}N_2O_2$ | $C_4H_7AsF_{12}N_2O_3$ |
| $M_r$ | 416.02 | 616.06 | 434.04 |
| Crystal system | Triclinic | Triclinic | Orthorhombic |
| Space group | *P*−1 | *P*−1 | *Pnma* |
| *T* / K | 100 | 100 | 100 |
| *a* / Å | 5.2815 (3) | 5.32051 (4) | 11.5349 (2) |
| *b* / Å | 10.1517 (6) | 10.45222 (9) | 14.0649 (3) |
| *c* / Å | 12.4911 (6) | 16.10170 (12) | 7.78994 (14) |
| α / ° | 108.936 (5) | 90.5740 (6) | 90 |
| β / ° | 93.107 (5) | 90.8760 (6) | 90 |
| γ / ° | 102.904 (5) | 103.0402 (7) | 90 |
| $V$ / $Å^3$ | 611.63 (6) | 872.16 (1) | 1263.82 (4) |
| *Z* | 2 | 2 | 4 |
| Radiation type | Cu Kα | Ag Kα, λ = 0.56087 Å | Cu Kα |
| μ / $mm^{-1}$ | 5.42 | 1.15 | 5.35 |
| Crystal size / mm | 0.06 × 0.05 × 0.03 | 0.7 × 0.5 × 0.42 | 0.28 × 0.17 × 0.15 |
| $T_{min}$, $T_{max}$ | 0.823, 0.933 | 0.184, 1.000 | 0.312, 1.000 |
| No. of measured, independent and observed [$I > 2\sigma(I)$] reflections | 17976, 2529, 1862 | 99695, 9247, 8391 | 35878, 1378, 1202 |
| $R_{int}$ | 0.102 | 0.038 | 0.095 |
| $\sin(\theta/\lambda)_{max}$ / $Å^{-1}$ | 0.629 | 0.892 | 0.630 |
| $R[F^2 > 2\sigma(F^2)]$, $wR(F^2)$, $S$ | 0.047, 0.125, 1.03 | 0.022, 0.055, 1.05 | 0.037, 0.085, 1.09 |
| No. of reflections | 2529 | 9247 | 1378 |
| No. of parameters | 210 | 319 | 124 |
| No. of restraints | / | / | / |
| H-atom treatment | All H-atom parameters refined | All H-atom parameters refined | All H-atom parameters refined |
| $\Delta\rho_{max}$, $\Delta\rho_{min}$ / e $Å^{-3}$ | 0.53, −0.98 | 0.55, −0.44 | 0.42, −0.53 |

Table S1. Continued …

| Compound | $CF_3C(OH)NH_2[AsF_6]\cdot XeF_2$ | $C_2F_5C(OH)NH_2[AsF_6]\cdot XeF_2$ | $C_3F_7C(OH)NH_2[AsF_6]\cdot XeF_2$ |
|---|---|---|---|
| Chemical formula | $C_2H_3AsF_{11}NOXe$ | $C_3H_3AsF_{13}NOXe$ | $C_4H_3AsF_{15}NOXe$ |
| $M_r$ / g mol$^{-1}$ | 472.27 | 522.28 | 572.29 |
| Crystal system | Monoclinic | Orthorhombic | Orthorhombic |
| Space group | $P2_1/n$ | *Aea*2 | *Pnna* |
| *T* / K | 100 | 100 | 100 |
| *a* / Å | 7.41785 (9) | 8.67561 (10) | 8.62011 (14) |
| *b* / Å | 9.84875 (11) | 31.0125 (4) | 35.5418 (5) |
| *c* / Å | 14.90113 (17) | 8.65174 (9) | 8.71910 (12) |
| α / ° | 90 | 90 | 90 |
| β / ° | 99.4517 (11) | 90 | 90 |
| γ / ° | 90 | 90 | 90 |
| *V* / Å$^3$ | 1073.85 (2) | 2327.77 (4) | 2671.31 (7) |
| *Z* | 4 | 8 | 8 |
| Radiation type | Ag Kα, λ = 0.56087 Å | Ag Kα, λ = 0.56087 Å | Ag Kα, λ = 0.56087 Å |
| μ / mm$^{-1}$ | 3.39 | 3.15 | 2.76 |
| Crystal size / mm | 0.24 × 0.14 × 0.08 | 0.27 × 0.17 × 0.07 | 0.37 × 0.28 × 0.05 |
| $T_{min}$, $T_{max}$ | 0.504, 1.000 | 0.458, 1.000 | 0.196, 1.000 |
| No. of measured, independent and observed [$I > 2\sigma(I)$] reflections | 100779, 8994, 7982 | 111431, 6484, 5368 | 133848, 5099, 4461 |
| $R_{int}$ | 0.095 | 0.045 | 0.053 |
| $\sin(\theta/\lambda)_{max}$ / Å$^{-1}$ | 1.000 | 0.912 | 0.769 |
| $R[F^2 > 2\sigma(F^2)]$, $wR(F^2)$, $S$ | 0.025, 0.067, 1.03 | 0.018, 0.038, 1.04 | 0.026, 0.066, 1.14 |
| No. of reflections | 8994 | 6484 | 5099 |
| No. of parameters | 194 | 197 | 221 |
| No. of restraints | 45 | 1 | 2 |
| H-atom treatment | All H-atom parameters refined | All H-atom parameters refined | All H-atom parameters refined |
| $\Delta\rho_{max}$, $\Delta\rho_{min}$ / e Å$^{-3}$ | 1.59, −1.95 | 0.50, −0.41 | 1.62, −1.16 |

$C_2F_5C(OH)NH_2[AsF_6]\cdot XeF_2$ was refined as a twin: Twin law: 0 0 1, 0 −1 0, 1 0 0 −4; BASF: 0.099 (11); 0.357 (10); 0.121 (11)

**Table S2**. C=O, C−N, (O)C−C bond lengths (Å) and N−C−O angles (°)

| Compound | C=O | C−N | (O)C−C | N−C−O |
|---|---|---|---|---|
| $CF_3CONH_2$[1] | 1.2304 (12) | 1.3164 (13) | 1.5395 (14) | 126.71 (9) |
| $C_2F_5CONH_2$ | 1.2323 (19) | 1.317 (2) | 1.536 (2) | 125.71 (16) |
| $C_3F_7CONH_2$ | 1.2293 (15) | 1.3162 (16) | 1.5480 (16) | 126.19 (11) |
| $CF_3C(OH)NH_2[AsF_6]$ | 1.282 (2)<br>1.2795 (19) | 1.279 (2)<br>1.281 (2) | 1.543 (2)<br>1.537 (2) | 127.51 (16)<br>127.50 (15) |
| $C_2F_5C(OH)NH_2[AsF_6]$ | 1.2821 (15) | 1.2772 (16) | 1.5307 (17) | 127.37 (11) |
| $C_3F_7C(OH)NH_2[AsF_6]$ | 1.2797 (14) | 1.2841 (16) | 1.5370 (15) | 127.81 (11) |
| $(CF_3CONH_2)_2H[AsF_6]$ | 1.283 (6)<br>1.247 (6) | 1.274 (7)<br>1.293 (7) | 1.516(7)<br>1.518 (7) | 125.8 (5)<br>124.1 (5) |
| $(C_3F_7CONH_2)_2H[AsF_6]$ | 1.2652 (9)<br>1.2459 (9) | 1.2942 (10)<br>1.3027 (10) | 1.5369 (10)<br>1.08 (10) | 126.65 (7)<br>123.81 (7) |
| $H_3O[AsF_6]·2CF_3CONH_2$ | 1.236 (4) | 1.304 (4) | 1.545(4) | 125.5 (3) |
| $CF_3C(OH)NH_2[AsF_6]·XeF_2$ | 1.2773 (15) | 1.2772 (16) | 1.5342 (15) | 127.37 (11) |
| $C_2F_5C(OH)NH_2[AsF_6]·XeF_2$ | 1.289 (2) | 1.279 (3) | 1.531 (3) | 126.4 (2) |
| $C_3F_7C(OH)NH_2[AsF_6]·XeF_2$ | 1.285 (3) | 1.280 (3) | 1.531 (3) | 126.1 (2) |

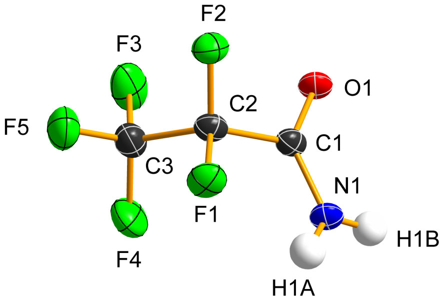


**Figure S1**. Asymmetric unit of the $C_2F_5CONH_2$ crystal structure. Displacement ellipsoids are shown at the 50 % probability level.

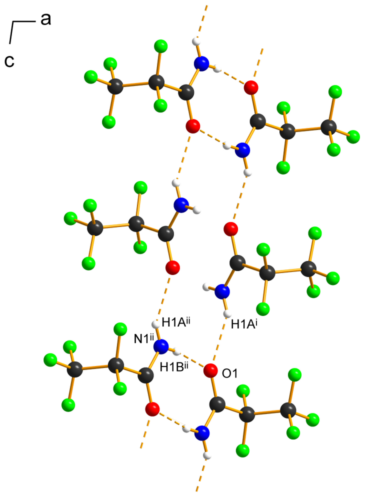


**Figure S2**. Hydrogen bonds (orange dashed lines; Table S3) in $C_2F_5CONH_2$.

**Table S3**. Hydrogen-bond geometry (Å, °) in $C_2F_5CONH_2$

| D–H···A | D–H | H···A | D···A | D–H···A |
|---|---|---|---|---|
| N1–H1A···O1$^{i}$ | 0.83 (3) | 2.10 (3) | 2.8396 (17) | 149 (2) |
| N1–H1B···O1$^{ii}$ | 0.88 (2) | 2.04 (2) | 2.912 (2) | 171 (2) |

Symmetry codes: (i) $x$, $-y + 1$, $z + 1/2$; (ii) $-x + 1/2$, $-y + 3/2$, $-z + 1$.

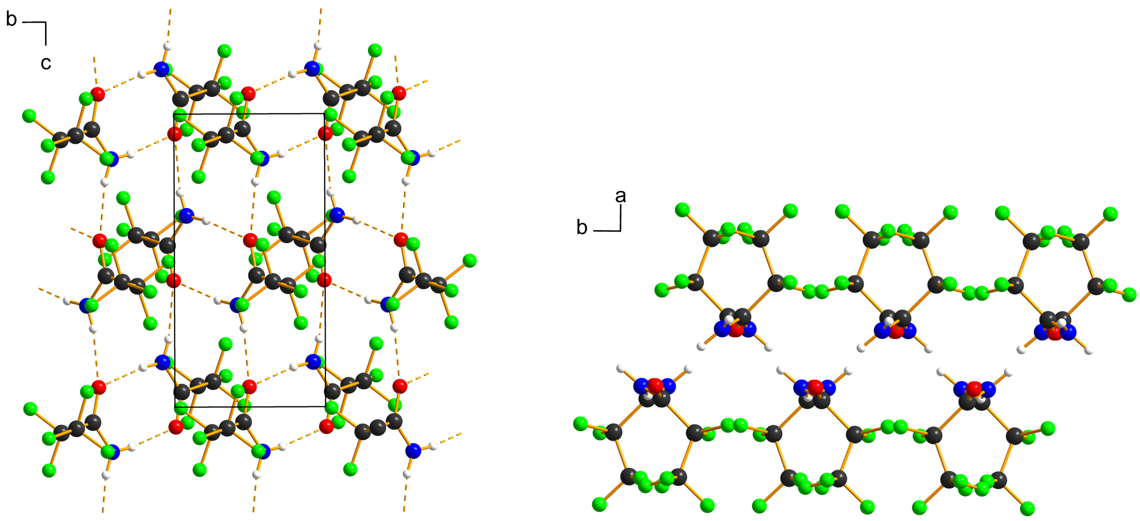


**Figure S3**. Hydrogen-bonded corrugated layer in the crystal structure of $C_2F_5CONH_2$ viewed along the *a*- (left) and *c*-crystallographic axis (right).

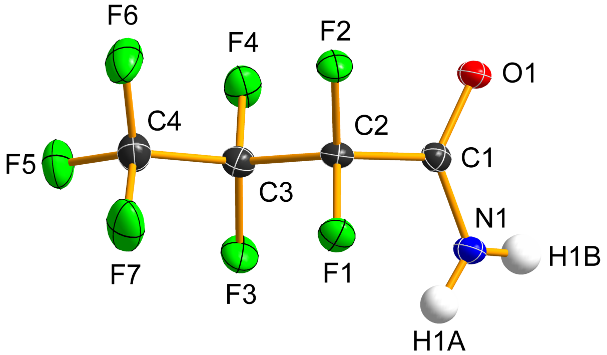


**Figure S4**. Asymmetric unit of the $C_3F_7CONH_2$ crystal structure. Displacement ellipsoids are shown at the 50 % probability level.

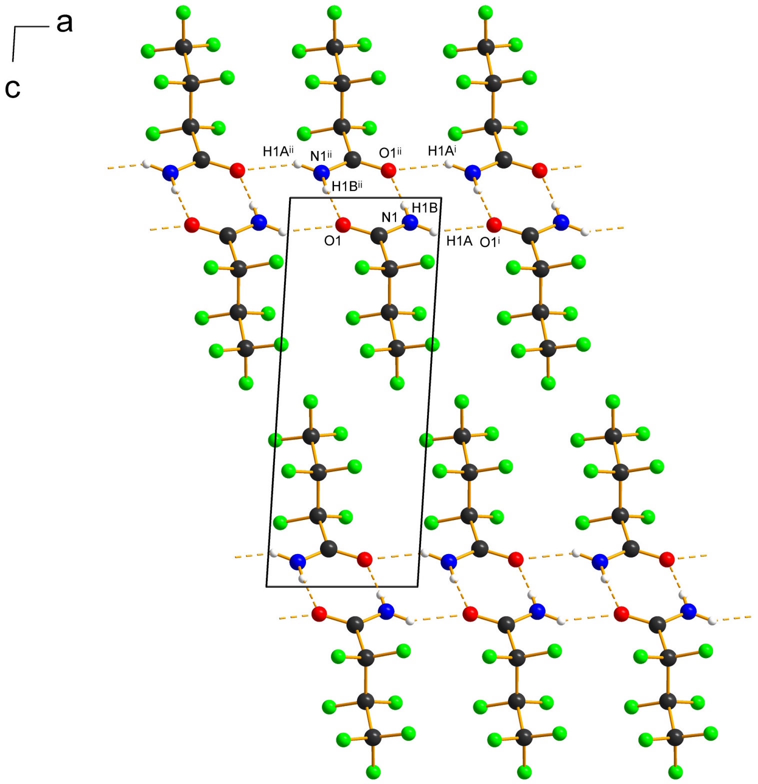


**Figure S5**. Hydrogen bonds (orange dashed lines; Table S4), packing diagram and unit cell of $C_3F_7CONH_2$ viewed along the *b*-crystallographic axis.

**Table S4**. Hydrogen-bond geometry (Å, °) in $C_3F_7CONH_2$

| D–H···A | D–H | H···A | D···A | D–H···A |
|---|---|---|---|---|
| N1—H1A···O1$^{i}$ | 0.839 (18) | 2.152 (18) | 2.8495 (14) | 140.4 (15) |
| N1–H1B···O1$^{ii}$ | 0.860 (19) | 2.075 (19) | 2.9313 (14) | 174.3 (16) |

Symmetry codes: (i) *x* + 1, *y*, *z*; (ii) −*x* + 1, −*y*, −*z*.

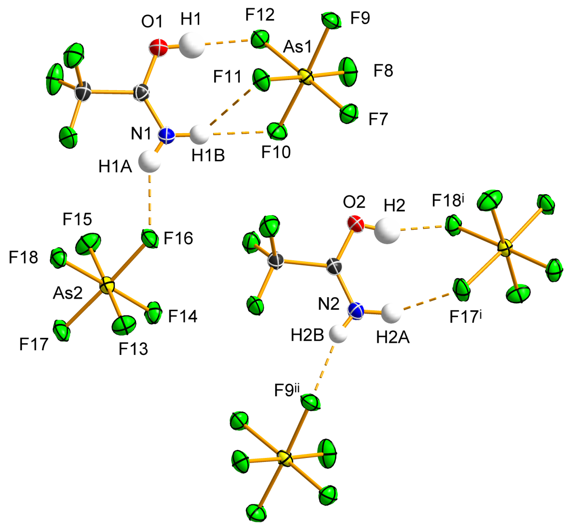


**Figure S6**. Expanded asymmetric unit of $CF_3C(OH)NH_2[AsF_6]$ and hydrogen bonds (orange dashed lines; Table S5). Displacement ellipsoids are shown at the 50 % probability level.

**Table S5**. Hydrogen-bond geometry (Å, °) in $CF_3C(OH)NH_2[AsF_6]$

| D–H···A | D–H | H···A | D···A | D–H···A |
|---|---|---|---|---|
| N1–H1A···F16 | 0.83 (3) | 2.10 (3) | 2.8420 (18) | 148 (3) |
| N1–H1B···F10 | 0.86 (3) | 2.07 (3) | 2.9262 (19) | 176 (2) |
| N1–H1B···F11 | 0.86 (3) | 2.55 (3) | 3.0797 (18) | 121 (2) |
| O1–H1···F12 | 0.87 (3) | 1.79 (3) | 2.6530 (18) | 179 (3) |
| N2–H2A···F17$^{i}$ | 0.91 (3) | 2.03 (3) | 2.8236 (18) | 145 (2) |
| N2–H2B···F9$^{ii}$ | 0.79 (3) | 2.06 (3) | 2.8330 (17) | 165 (3) |
| O2–H2···F18$^{i}$ | 0.84 (3) | 1.77 (3) | 2.5860 (16) | 163 (3) |

Symmetry codes: (i) *x*, −*y* + 1/2, *z* − 1/2; (ii) *x* − 1, *y*, *z*.

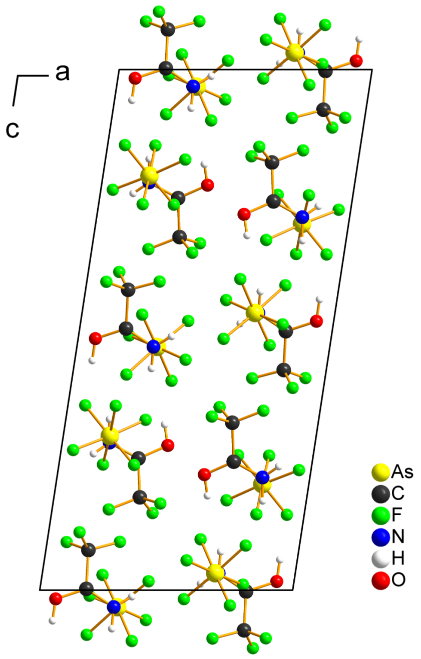


**Figure S7**. Packing diagram and the unit cell of the $CF_3C(OH)NH_2[AsF_6]$ crystal structure viewed along the *b*-crystallographic axis.

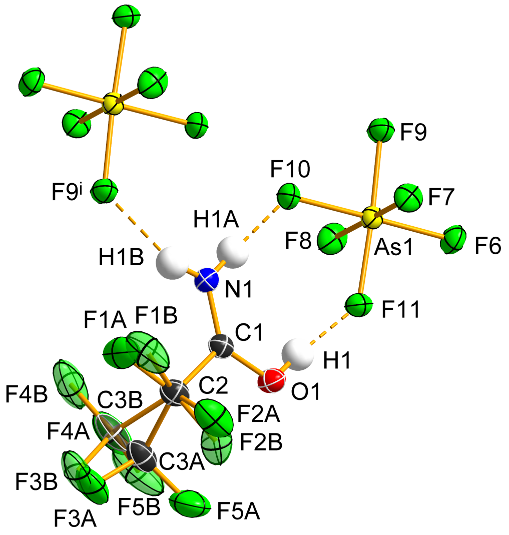


**Figure S8**. Expanded asymmetric unit of $C_2F_5C(OH)NH_2[AsF_6]$ and hydrogen bonds (orange dashed lines; Table S6). The minor component of the disordered $C_2F_5$ group (0.264 (3) : 0.736 (3)) is shown semitransparent, and all displacement ellipsoids are drawn at the 50 % probability level.

**Table S6**. Hydrogen-bond geometry (Å, °) in $C_2F_5C(OH)NH_2[AsF_6]$

| D–H⋯A | D–H | H⋯A | D⋯A | D–H⋯A |
|---|---|---|---|---|
| N1–H1A⋯F10 | 0.87 (2) | 1.97 (2) | 2.8309 (13) | 174 (2) |
| N1–H1B⋯F9$^{i}$ | 0.83 (2) | 2.03 (2) | 2.8316 (14) | 161.3 (19) |
| O1–H1⋯F11 | 0.882 (19) | 1.72 (2) | 2.6006 (12) | 172 (2) |

Symmetry codes: (i) −*x* + 1, −*y* + 1, −*z* + 2.

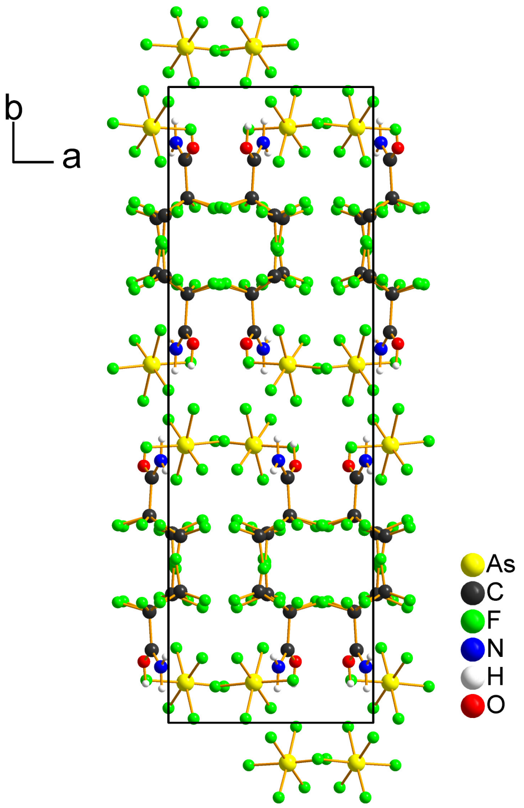


**Figure S9**. Packing diagram and unit cell of the $C_2F_5C(OH)NH_2[AsF_6]$ crystal structure viewed along the *c*-crystallographic axis.

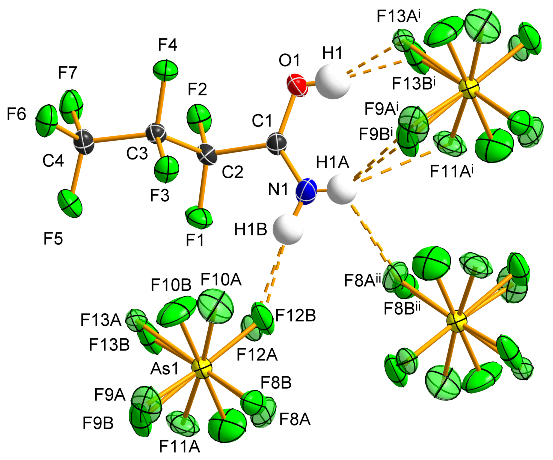


**Figure S10**. Expanded asymmetric unit of $C_3F_7C(OH)NH_2[AsF_6]$ and hydrogen bonds (orange dashed lines; Table S7). One component of the disordered $[AsF_6]^-$ anion (0.494 (4) : 0.506 (4)) is shown semitransparent, and all displacement ellipsoids are drawn at the 50 % probability level.

**Table S7**. Hydrogen-bond geometry (Å, °) in $C_3F_7C(OH)NH_2[AsF_6]$

| D–H···A | D—H | H···A | D···A | D—H···A |
|---|---|---|---|---|
| N1–H1A···F11A$^{i}$ | 0.79 (2) | 2.50 (2) | 3.125 (3) | 138 (2) |
| N1–H1A···F8A$^{ii}$ | 0.79 (2) | 2.25 (2) | 2.752 (3) | 122 (2) |
| N1–H1A···F9A$^{i}$ | 0.79 (2) | 2.51 (2) | 3.179 (5) | 144 (2) |
| N1–H1A···F9B$^{i}$ | 0.79 (2) | 2.03 (2) | 2.737 (4) | 148 (2) |
| N1–H1A···F8B$^{ii}$ | 0.79 (2) | 2.38 (2) | 2.840 (3) | 118.0 (19) |
| N1–H1B···F12A | 0.87 (2) | 2.17 (2) | 3.026 (3) | 168.1 (19) |
| N1–H1B···F12B | 0.87 (2) | 1.93 (2) | 2.782 (3) | 167 (2) |
| O1–H1···F13A$^{i}$ | 0.77 (3) | 1.81 (3) | 2.541 (3) | 157 (3) |
| O1–H1···F13B$^{i}$ | 0.77 (3) | 1.80 (3) | 2.557 (3) | 165 (3) |

Symmetry codes: (i) $x, y - 1, z$; (ii) $-x + 2, -y + 1, -z + 1$.

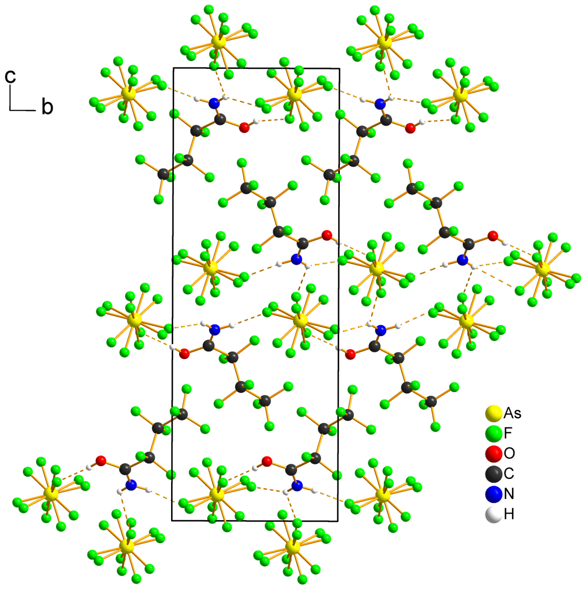


**Figure S11**. Packing diagram and unit cell of the $C_3F_7C(OH)NH_2[AsF_6]$ crystal structure viewed along the *a*-crystallographic axis. Hydrogen bonds are shown as orange dashed lines.

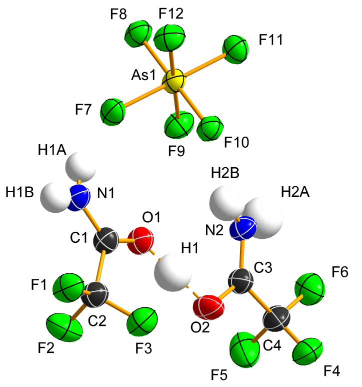


**Figure S12**. Asymmetric unit of $(CF_3CONH_2)_2H[AsF_6]$. Displacement ellipsoids are drawn at the 50 % probability level. Hydrogen bonds are shown as orange dashed lines.

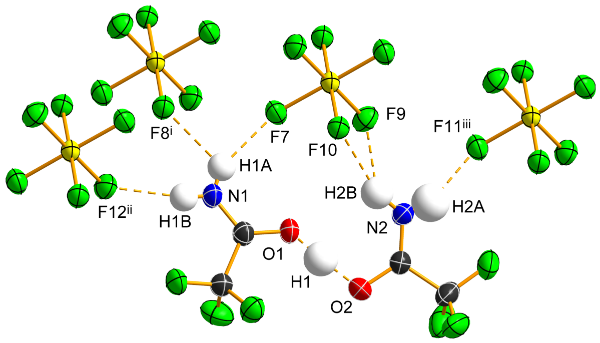


**Figure S13**. Hydrogen bonds in $(CF_3CONH_2)_2H[AsF_6]$ (orange dashed lines; Table S8). Displacement ellipsoids are drawn at the 50 % probability level. Symmetry codes are given in Table S8.

**Table S8**. Hydrogen-bond geometry (Å, °) in $(CF_3CONH_2)_2H[AsF_6]$

| D–H···A | D–H | H···A | D···A | D–H···A |
|---|---|---|---|---|
| N1–H1A···F7 | 0.82 (8) | 2.16 (8) | 2.924 (6) | 154 (7) |
| N1–H1A···F8$^{i}$ | 0.82 (8) | 2.38 (7) | 2.894 (5) | 121 (6) |
| N1–H1B···F12$^{ii}$ | 0.77 (8) | 2.16 (8) | 2.923 (5) | 170 (7) |
| O1–H1···O2 | 1.13 (9) | 1.31 (9) | 2.426 (5) | 170 (8) |
| N2–H2B···F9 | 0.93 (8) | 2.54 (7) | 3.005 (5) | 111 (5) |
| N2–H2B···F10 | 0.93 (8) | 2.11 (8) | 3.001 (5) | 161 (6) |
| N2–H2A···F6 | 0.81 (10) | 2.24 (10) | 2.644 (5) | 111 (8) |
| N2–H2A···F11$^{iii}$ | 0.81 (10) | 2.09 (10) | 2.841 (5) | 153 (10) |

Symmetry codes: (i) $-x + 2, -y + 2, -z + 1$; (ii) $-x + 1, -y + 2, -z + 1$; (iii) $-x + 2, -y + 1, -z + 1$.

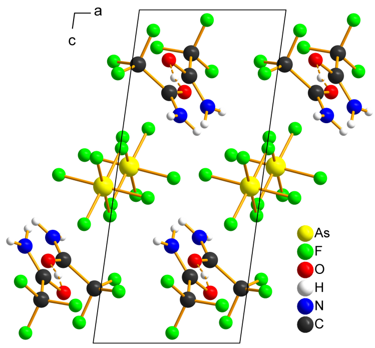


**Figure S14**. Packing diagram and unit cell of the $(CF_3CONH_2)_2H[AsF_6]$ crystal structure viewed along the *b*-crystallographic axis.

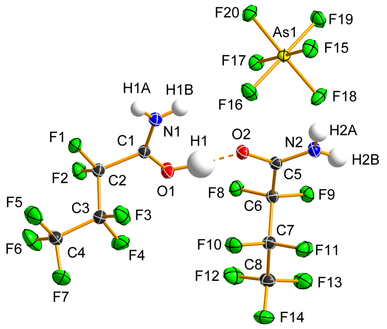


**Figure S15**. Asymmetric unit of $(C_3F_7CONH_2)_2H[AsF_6]$. Displacement ellipsoids are drawn at the 50 % probability level. Hydrogen bonds are shown as orange dashed lines.

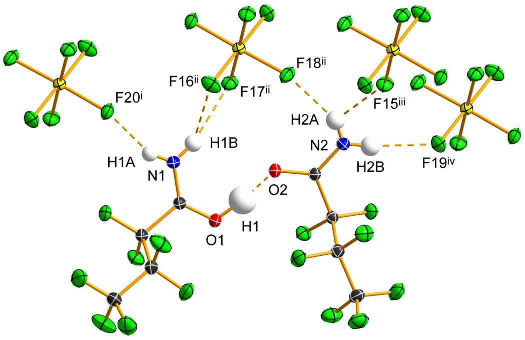


**Figure S16**. Hydrogen bonds in $(C_3F_7CONH_2)_2H[AsF_6]$ (orange dashed lines; Table S9). Displacement ellipsoids are drawn at the 50 % probability level. Symmetry codes are given in Table S9.

**Table S9**. Hydrogen-bond geometry (Å, °) in $(C_3F_7CONH_2)_2H[AsF_6]$

| D–H⋯A | D–H | H⋯A | D⋯A | D–H⋯A |
|---|---|---|---|---|
| N1–H1A⋯F20$^{i}$ | 0.806 (15) | 2.103 (14) | 2.8485 (9) | 153.9 (14) |
| N1–H1B⋯F16$^{ii}$ | 0.844 (15) | 2.434 (14) | 3.0039 (9) | 125.5 (12) |
| N1–H1B⋯F17$^{ii}$ | 0.844 (15) | 2.295 (15) | 3.1046 (9) | 161.0 (13) |
| O1–H1⋯O2 | 1.06 (2) | 1.36 (2) | 2.4174 (9) | 172 (2) |
| N2–H2A⋯F15$^{iii}$ | 0.815 (17) | 2.349 (16) | 2.8168 (9) | 117.2 (14) |
| N2–H2A⋯F18$^{ii}$ | 0.815 (17) | 2.155 (17) | 2.9036 (9) | 152.7 (15) |
| N2–H2B⋯F19$^{iv}$ | 0.857 (17) | 2.096 (17) | 2.9464 (9) | 171.7 (15) |

Symmetry codes: (i) $-x, -y + 1, -z + 1$; (ii) $x - 1, y, z$; (iii) $-x, -y, -z + 1$; (iv) $-x + 1, -y, -z + 1$.

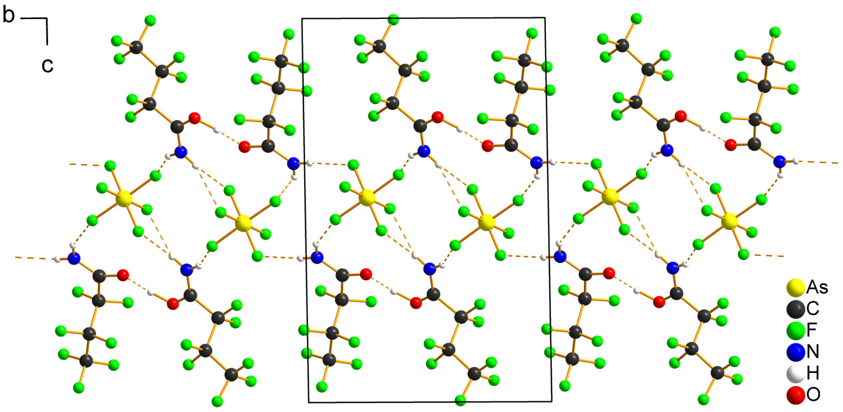


**Figure S17**. Packing diagram and unit cell of the $(C_3F_7CONH_2)_2H[AsF_6]$ crystal structure viewed along the *a*-crystallographic axis.

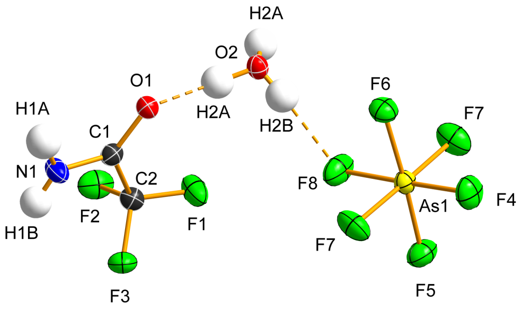


**Figure S18**. Asymmetric unit of $H_3O[AsF_6]\cdot2CF_3C(O)NH_2$. Displacement ellipsoids are drawn at the 50 % probability level. Hydrogen bonds are shown as orange dashed lines.

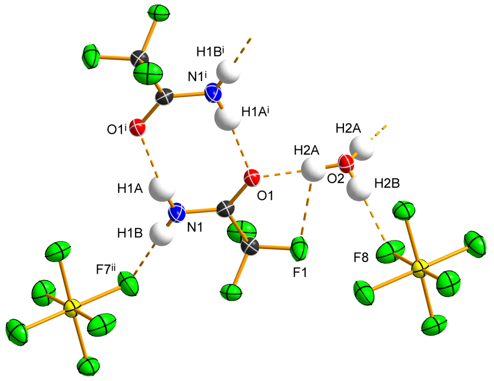


**Figure S19**. Hydrogen bonds in $H_3O[AsF_6]\cdot2CF_3CONH_2$ (orange dashed lines; Table S10). Displacement ellipsoids are drawn at the 50 % probability level. Symmetry codes are given in Table S10.

**Table S10**. Hydrogen bond geometry (Å, °) in $H_3O[AsF_6]\cdot2CF_3CONH_2$

| D–H···A | D–H | H···A | D···A | D–H···A |
|---|---|---|---|---|
| N1–H1A···O1$^{i}$ | 0.93 (5) | 2.04 (5) | 2.959 (4) | 171 (4) |
| N1–H1B···F7$^{ii}$ | 0.82 (5) | 2.27 (5) | 3.065 (4) | 166 (4) |
| O2–H2B···F8 | 0.77 (8) | 1.89 (8) | 2.657 (5) | 173 (7) |
| O2–H2A···F1 | 0.89 (5) | 2.51 (4) | 2.967 (4) | 112 (3) |
| O2–H2A···O1 | 0.89 (5) | 1.65 (5) | 2.525 (3) | 167 (4) |

Symmetry codes: (i) $-x + 1, -y + 1, -z$; (ii) $-x + 1, y + 1/2, -z + 1$.

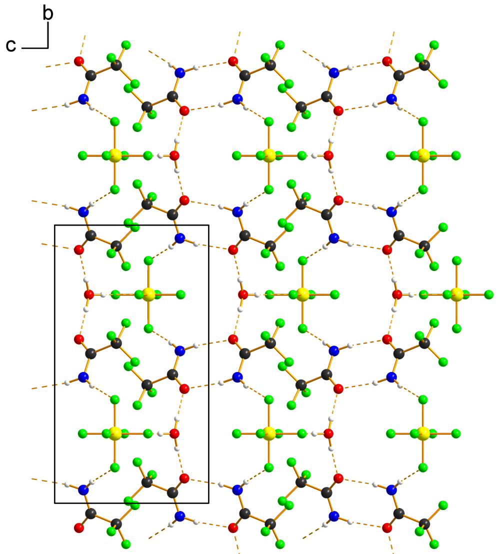


**Figure S20**. Packing diagram and unit cell of the $H_3O[AsF_6]\cdot2CF_3CONH_2$ crystal structure viewed along the *a*-crystallographic axis.

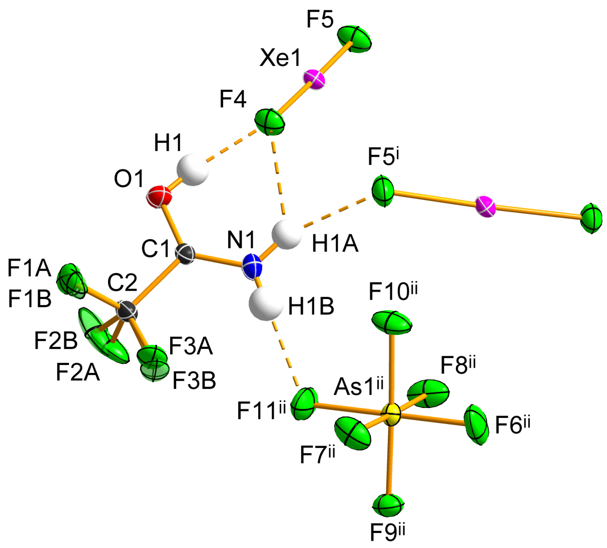


**Figure S21**. Expanded asymmetric unit of $CF_3C(OH)NH_2[AsF_6]\cdot XeF_2$ and hydrogen bonds (orange dashed lines; Table S11). The minor component of the disordered $CF_3$ group (0.46 (2) : 0.54 (2)) is shown semitransparent, and all displacement ellipsoids are drawn at the 50 % probability level. Symmetry codes are given in Table S11.

**Table S11**. Hydrogen-bond geometry (Å, °) in $CF_3C(OH)NH_2[AsF_6]\cdot XeF_2$

| D–H···A | D–H | H···A | D···A | D–H···A |
|---|---|---|---|---|
| N1–H1A···F4 | 0.88 (3) | 2.51 (3) | 3.0894 (16) | 124 (2) |
| N1–H1A···F5$^{i}$ | 0.88 (3) | 1.98 (3) | 2.7865 (15) | 151 (3) |
| N1–H1A···F9 | 0.88 (3) | 2.64 (3) | 3.0594 (15) | 111 (2) |
| N1–H1B···F11$^{ii}$ | 0.77 (3) | 2.05 (3) | 2.8270 (18) | 176 (3) |
| O1–H1···F4 | 0.85 (3) | 1.71 (3) | 2.5467 (14) | 171 (3) |

Symmetry codes: (i) −*x* + 3/2, *y* − 1/2, −*z* + 1/2; (ii) −*x* + 1/2, *y* − 1/2, −*z* + 1/2.

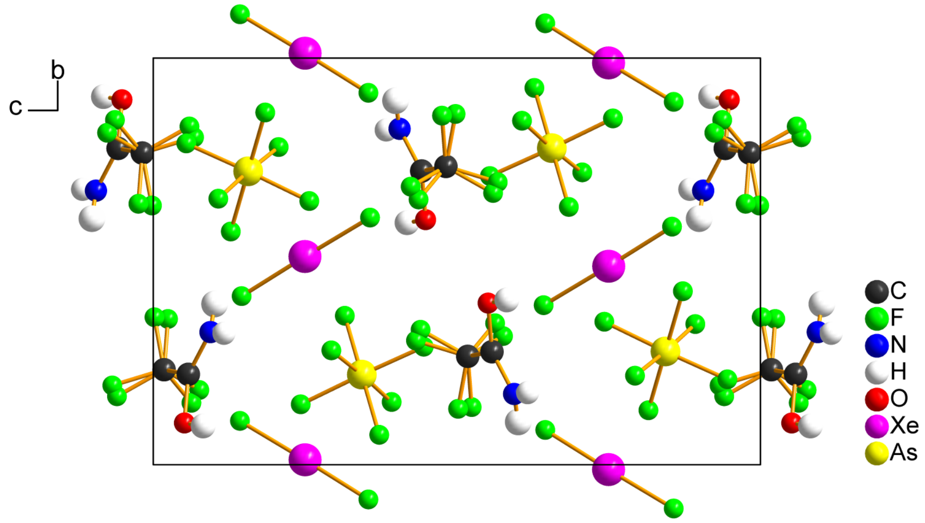


**Figure S22**. Packing diagram and unit cell of the $CF_3C(OH)NH_2[AsF_6]\cdot XeF_2$ crystal structure viewed along the *a*-crystallographic axis.

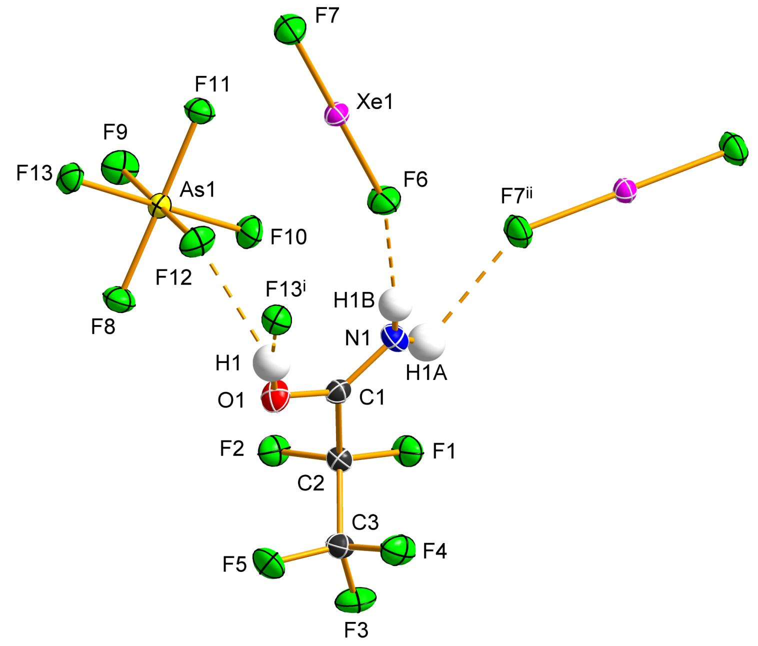


**Figure S23**. Expanded asymmetric unit of $C_2F_5C(OH)NH_2[AsF_6]\cdot XeF_2$ and hydrogen bonds (orange dashed lines; Table S12). The $[AsF_6]^-$ anion containing F13$^i$ atom is omitted for clarity. Displacement ellipsoids are shown at the 50 % probability level.

**Table S12***.* Hydrogen-bond geometry (Å, °) in $C_2F_5C(OH)NH_2[AsF_6]\cdot XeF_2$

| D–H···A | D–H | H···A | D···A | D–H···A |
|---|---|---|---|---|
| O1–H1···F12 | 0.83 (3) | 2.33 (3) | 2.872 (2) | 124 (3) |
| O1–H1···F13$^i$ | 0.83 (3) | 1.90 (3) | 2.690 (2) | 159 (3) |
| N1–H1B···F7$^{ii}$ | 0.69 (4) | 2.36 (4) | 2.688 (3) | 111 (4) |
| N1–H1A···F6 | 0.79 (3) | 1.97 (4) | 2.729 (3) | 162 (4) |

Symmetry codes: (i) $-x + 1/2$, $y$, $z + 1/2$; (ii) $x + 1/2$, $-y + 1$, $z + 1/2$.

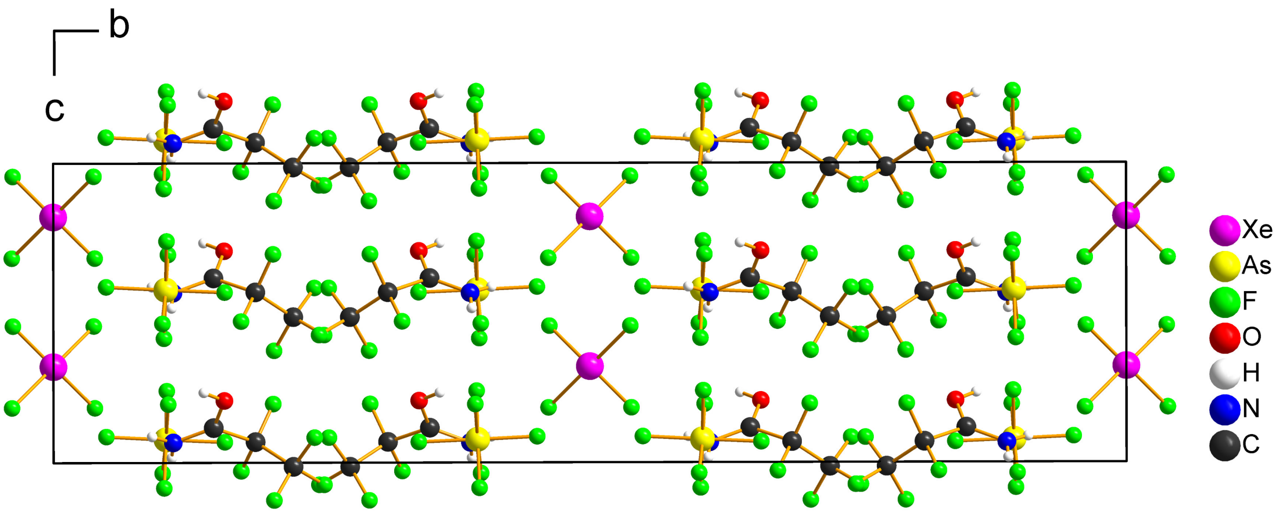


**Figure S24**. Packing diagram and the unit cell of $C_2F_5C(OH)NH_2[AsF_6]\cdot XeF_2$ crystal structure viewed along the *a*-crystallographic axis.

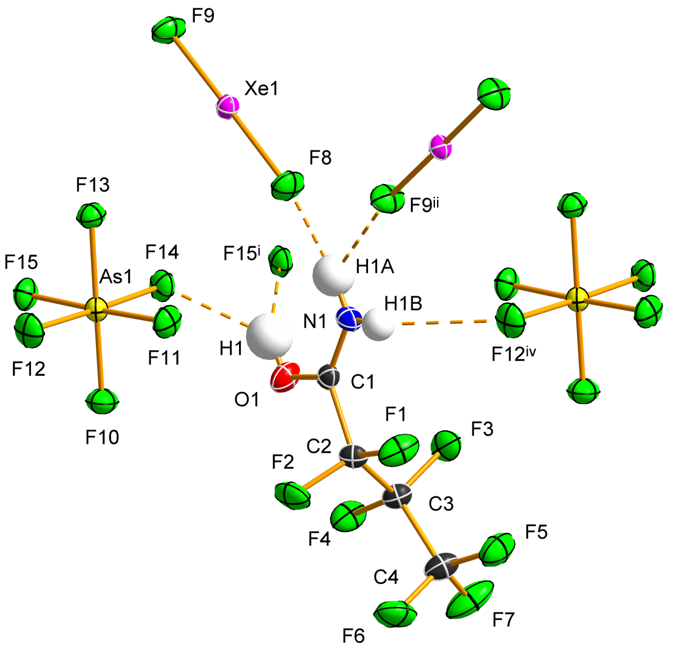


**Figure S25**. Expanded asymmetric unit of $C_3F_7C(OH)NH_2[AsF_6]\cdot XeF_2$ and hydrogen bonds (orange dashed lines; Table S13). The $[AsF_6]^-$ anion containing $F15^i$ atom is omitted for clarity. Displacement ellipsoids are shown at the 50 % probability level.

**Table S13.** Hydrogen-bond geometry (Å, °) in $C_3F_7C(OH)NH_2[AsF_6]\cdot XeF_2$.

| D–H···A | D–H | H···A | D···A | D–H···A |
|---|---|---|---|---|
| O1–H1···F14 | 0.79 (5) | 2.11 (5) | 2.740 (3) | 136 (5) |
| O1–H1···F15$^{i}$ | 0.79 (5) | 2.17 (5) | 2.770 (3) | 133 (5) |
| N1–H1A···F8 | 0.842 (19) | 1.90 (2) | 2.737 (3) | 169 (5) |
| N1–H1A···F9$^{ii}$ | 0.842 (19) | 2.36 (5) | 2.692 (3) | 104 (4) |
| N1–H1B···F11$^{iii}$ | 0.849 (18) | 2.58 (3) | 3.334 (3) | 148 (3) |
| N1–H1B···F12$^{iv}$ | 0.849 (18) | 2.49 (3) | 3.229 (3) | 146 (3) |

Symmetry codes: (i) *x* + 1/2, *y*, −*z* + 1; (ii) *x* + 1/2, −*y* + 1/2, *z* + 1/2; (iii) *x* + 1/2, *y*, −*z* + 2; (iv) *x* + 1, *y*, *z*.

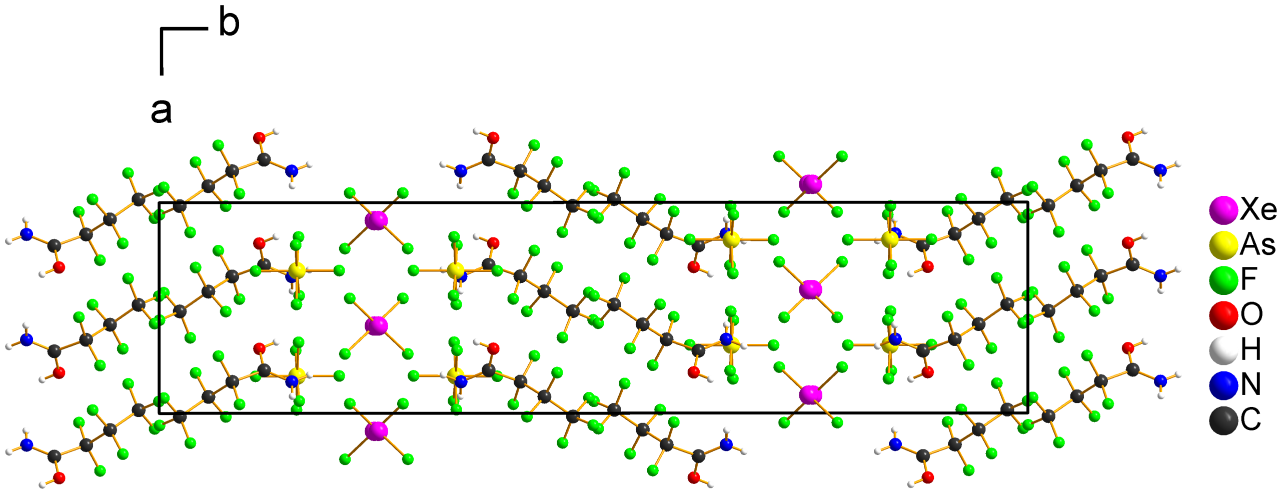


**Figure S26**. Packing diagram and unit cell of the $C_3F_7C(OH)NH_2[AsF_6]\cdot XeF_2$ crystal structure viewed along the *c*-crystallographic axis.

## 2. Low-temperature Raman spectra

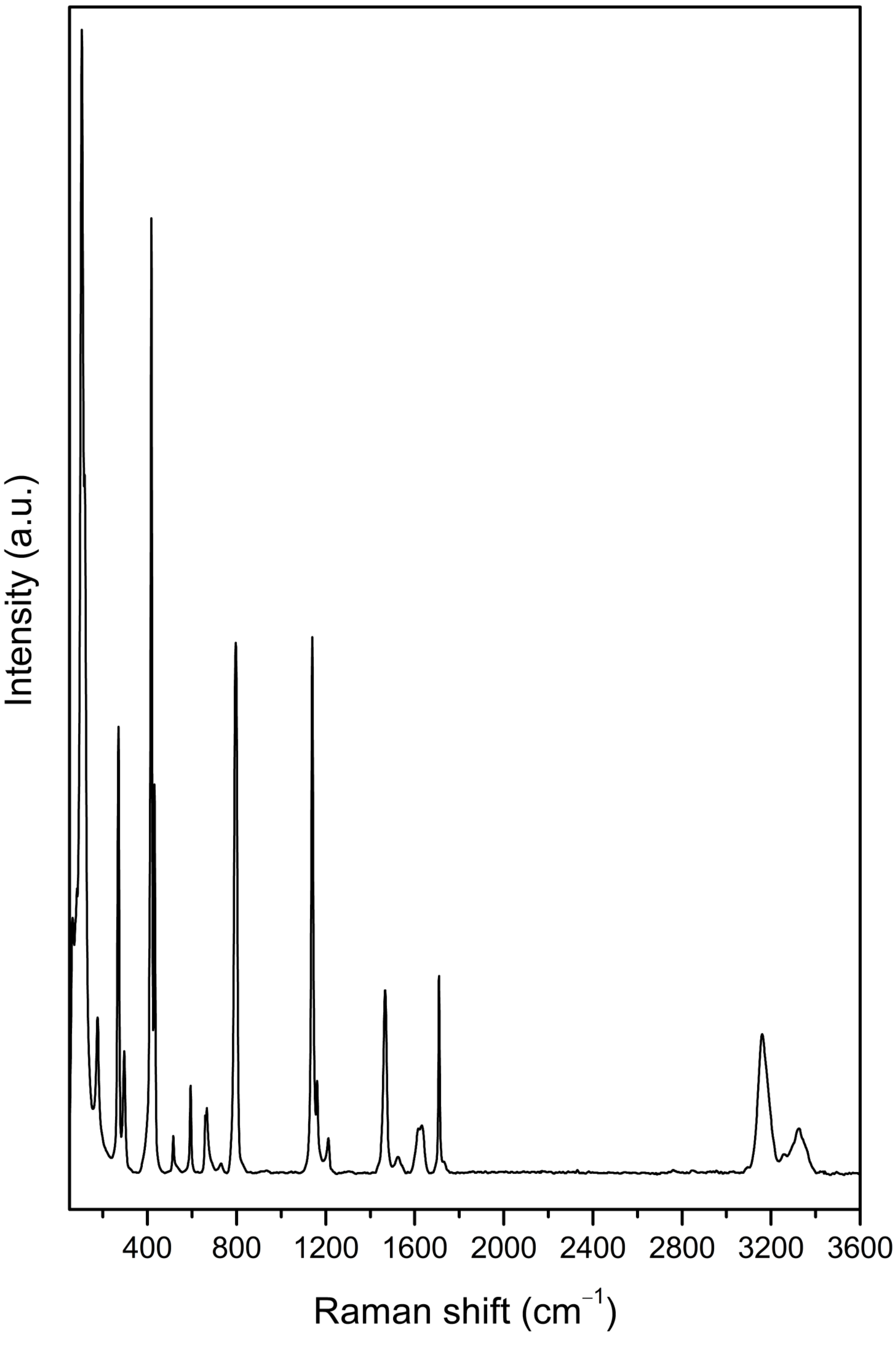


**Figure S27**. Raman spectrum of $CF_3CONH_2$ (−90 °C).

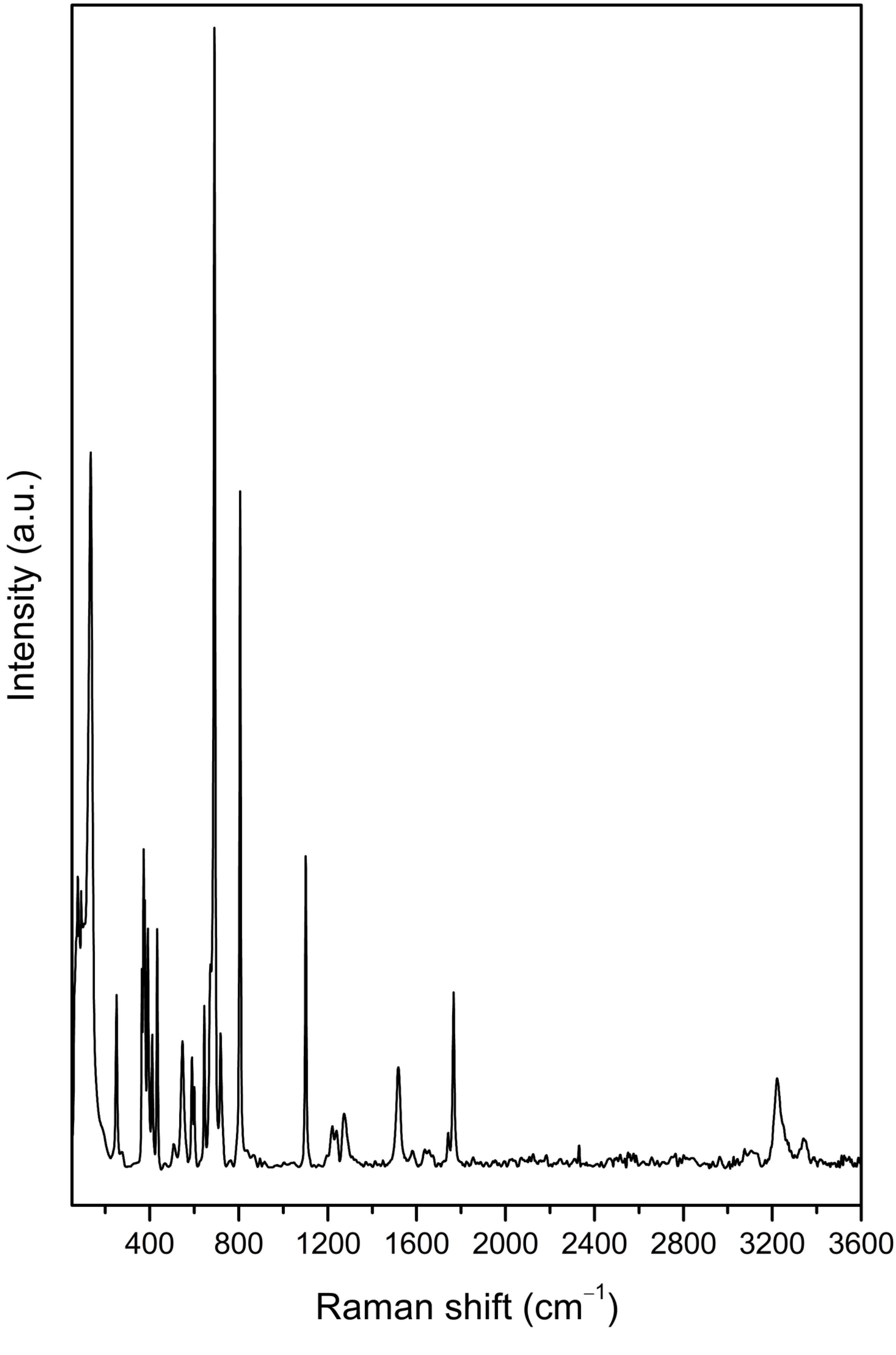


**Figure S28**. Raman spectrum of $CF_3C(OH)NH_2[AsF_6]$ (−100 °C).

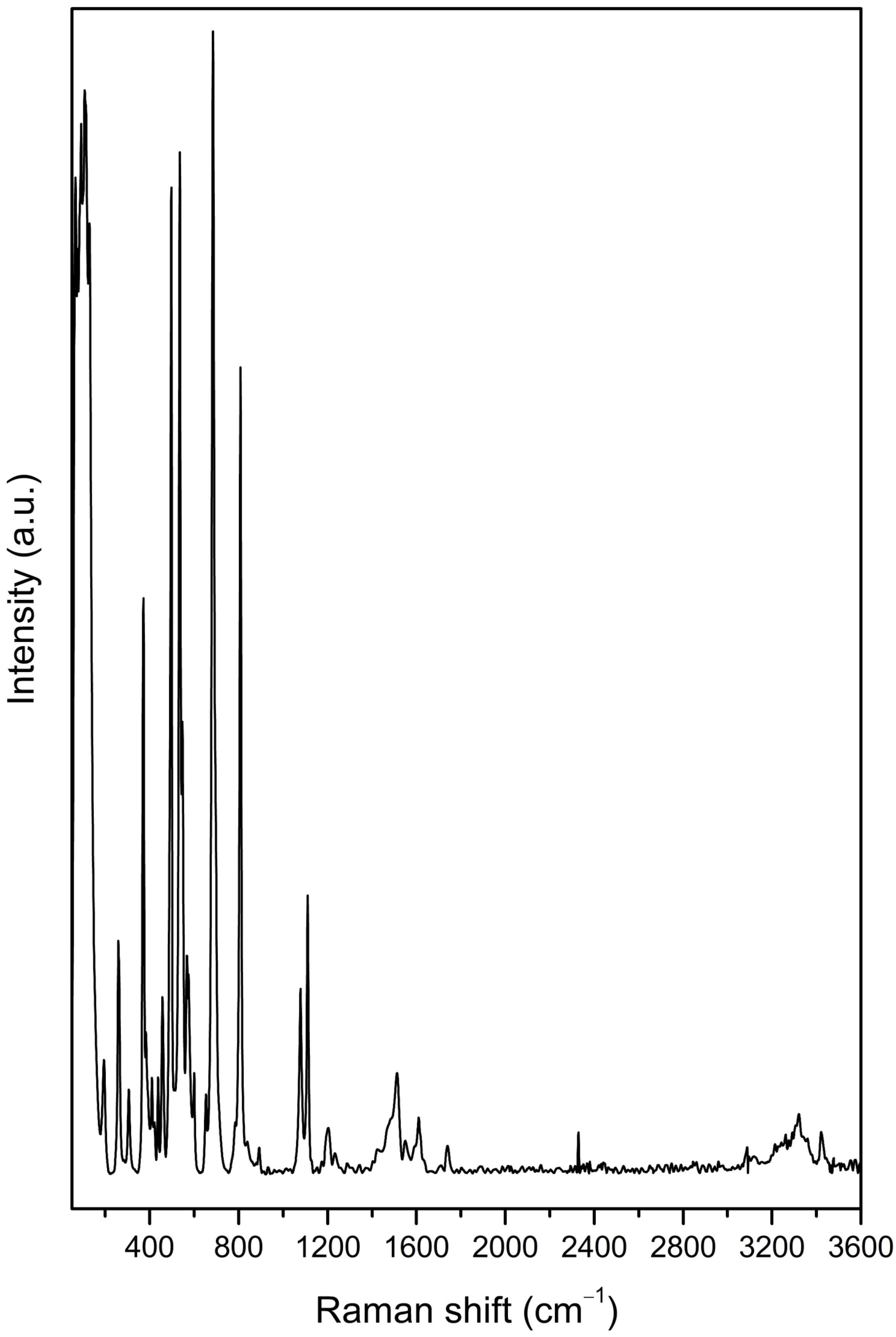


**Figure S29**. Raman spectrum of $CF_3C(OH)NH_2[AsF_6]\cdot XeF_2$ (−90 °C). Peak at 2327 cm$^{-1}$ corresponds to $N_2$ from the nitrogen stream.

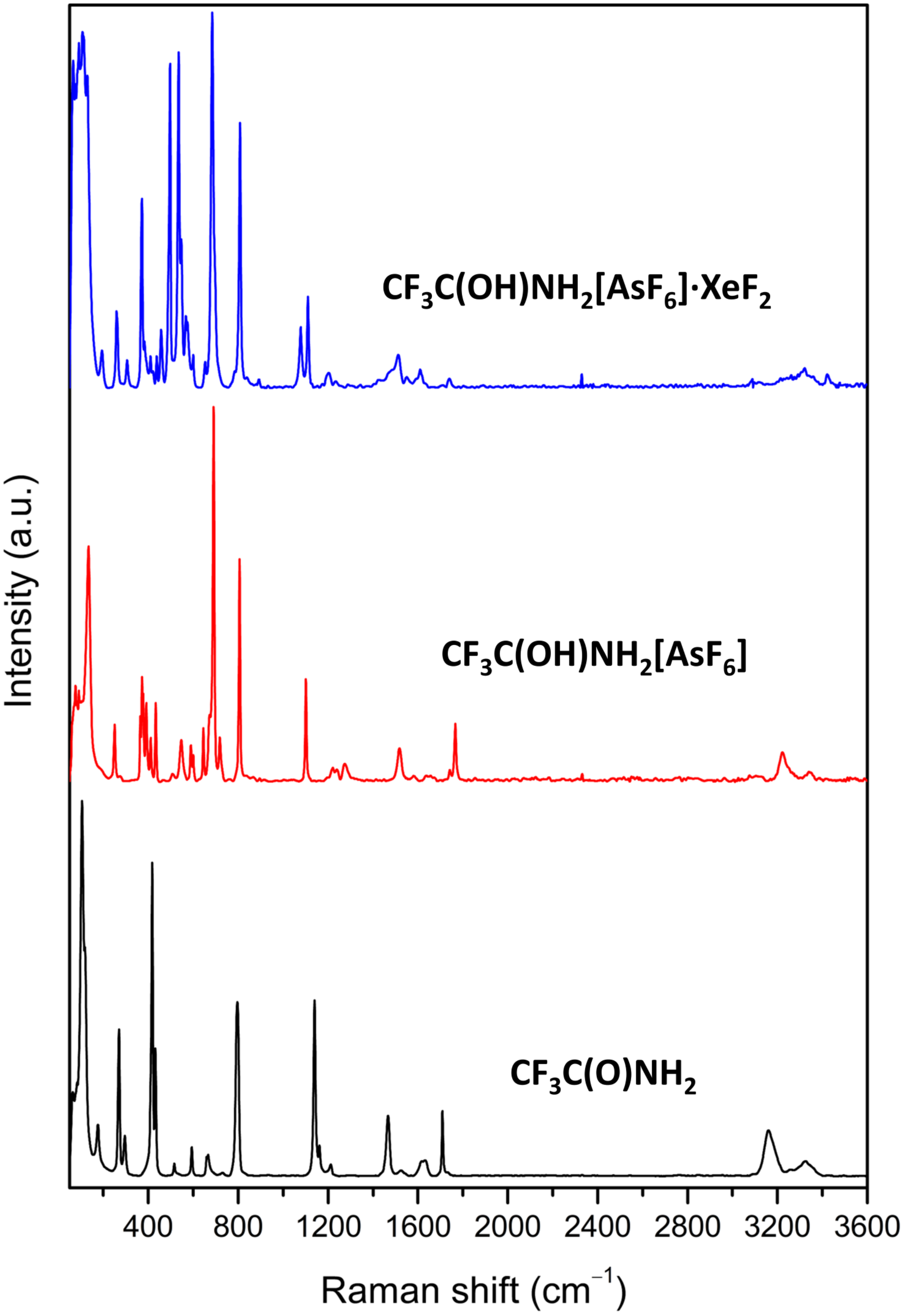


Figure S30. Comparison of Raman spectra of $CF_3CONH_2$, $CF_3C(OH)NH_2[AsF_6]$, and $CF_3C(OH)NH_2[AsF_6]\cdot XeF_2$.

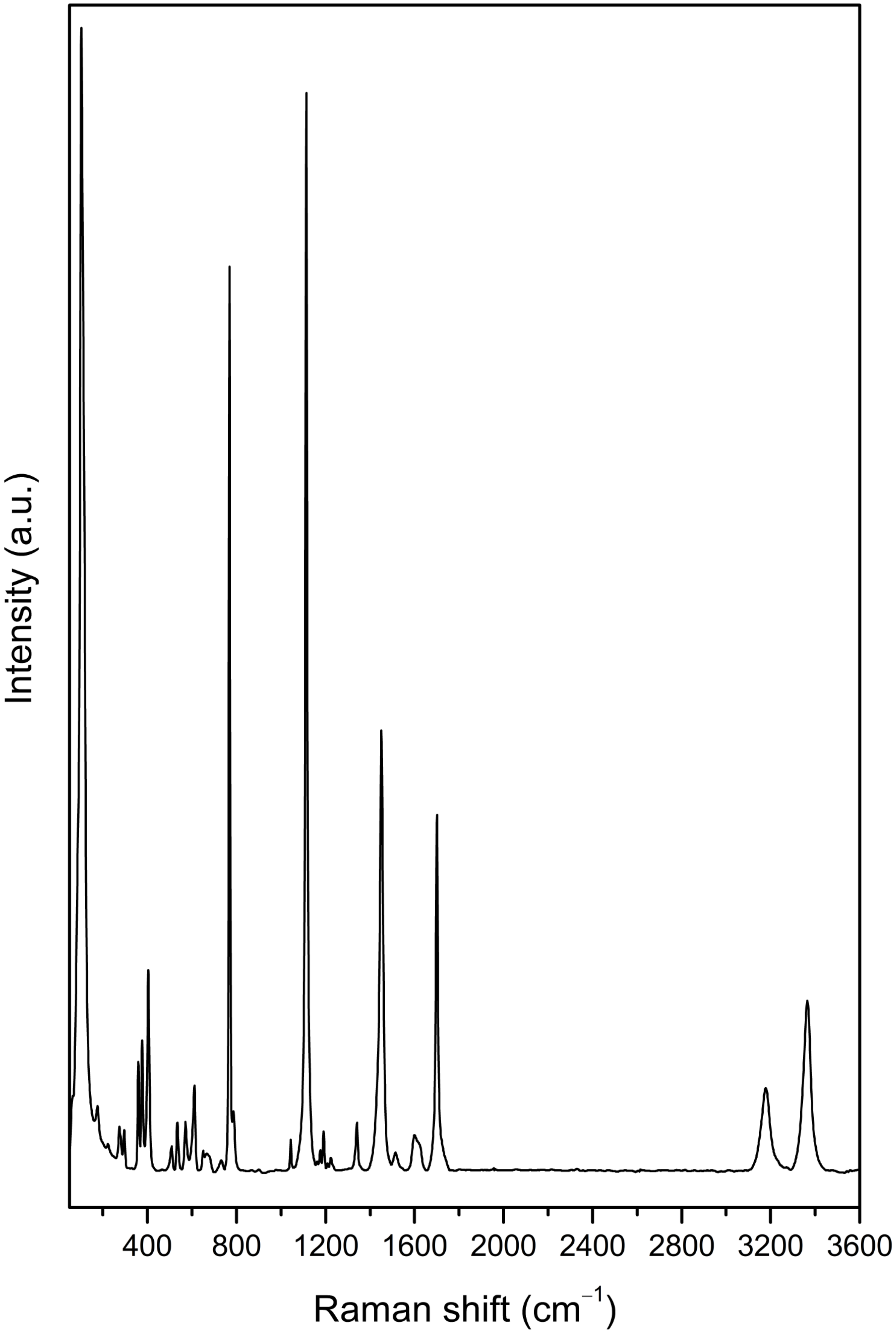


**Figure S31**. Raman spectrum of $C_2F_5CONH_2$ (−90 °C).

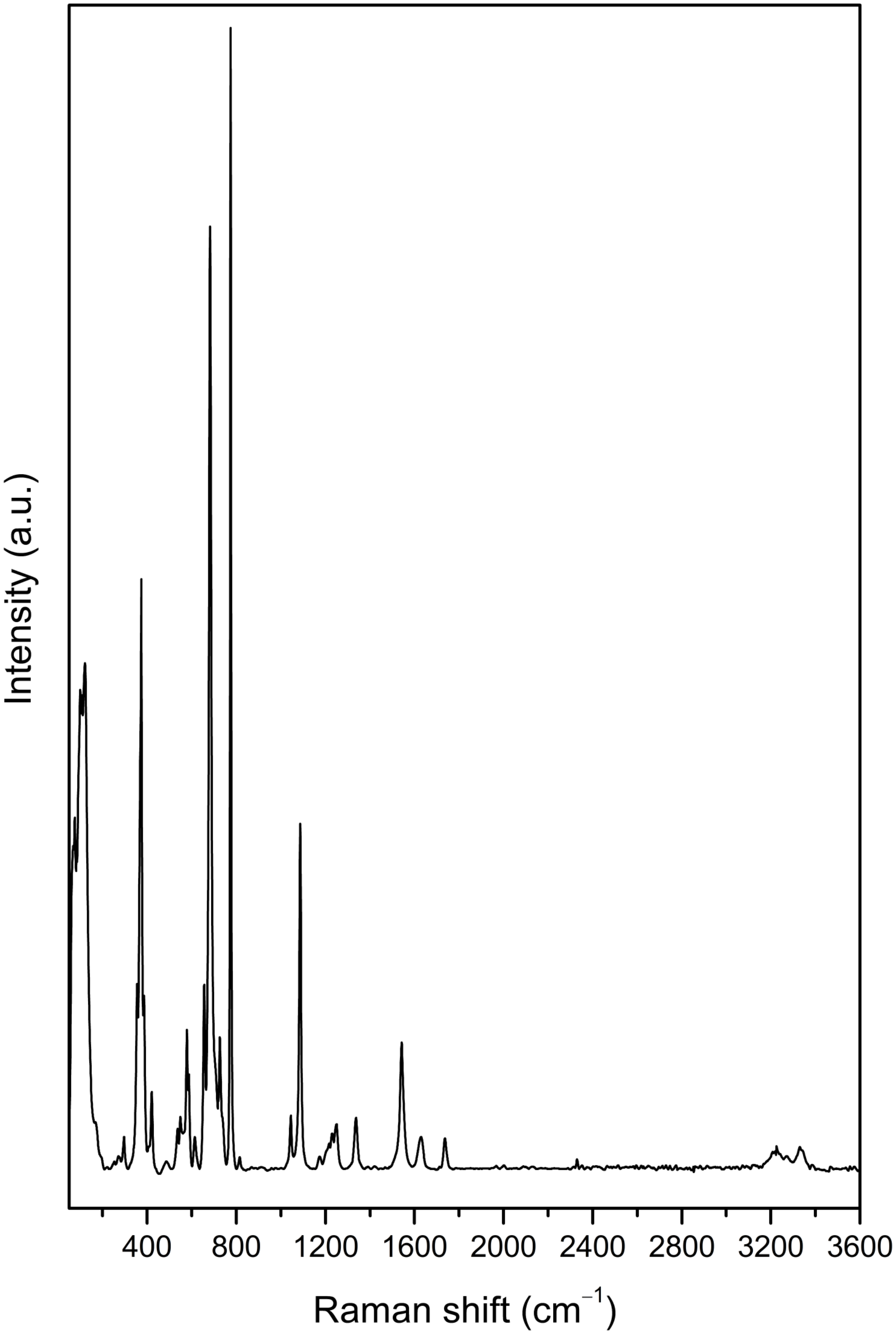


**Figure S32**. Raman spectrum of $C_2F_5C(OH)NH_2[AsF_6]$ (−50 °C).

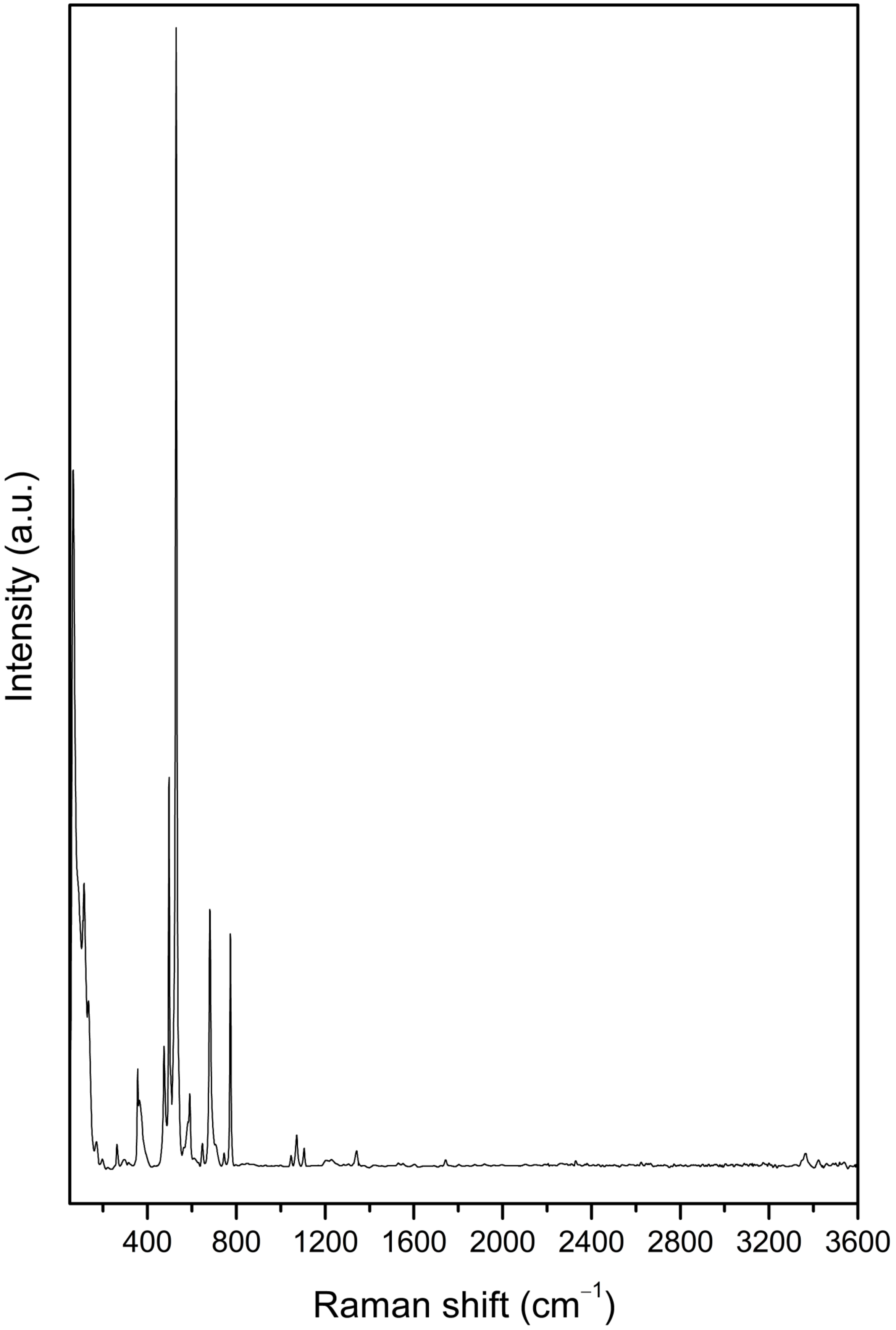


**Figure S33**. Raman spectrum of $C_2F_5C(OH)NH_2[AsF_6]{\cdot}XeF_2$ (−90 °C).

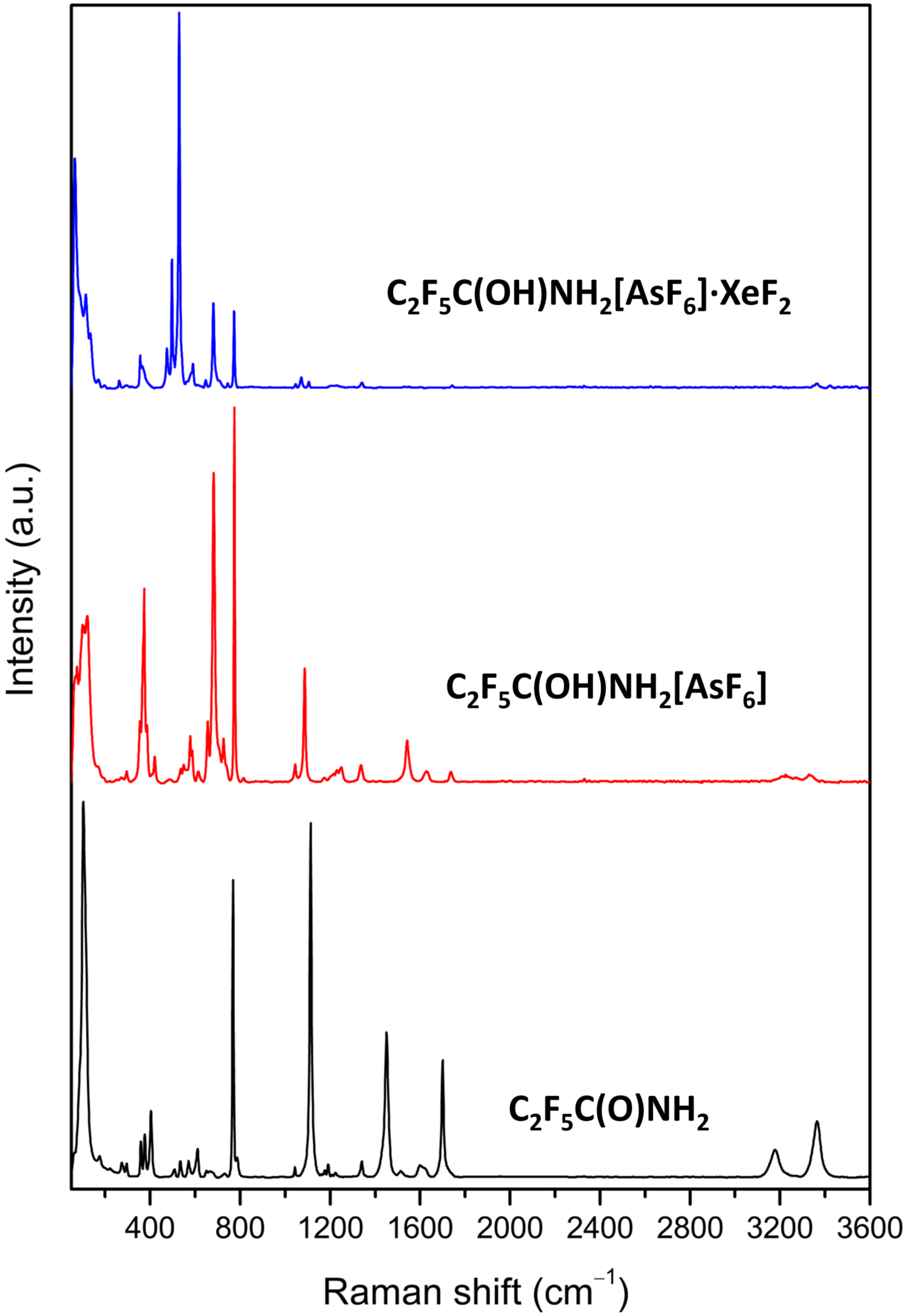


**Figure S34**. Raman spectra of $C_2F_5CONH_2$, $C_2F_5C(OH)NH_2[AsF_6]$, and $C_2F_5C(OH)NH_2[AsF_6]{\cdot}XeF_2$.

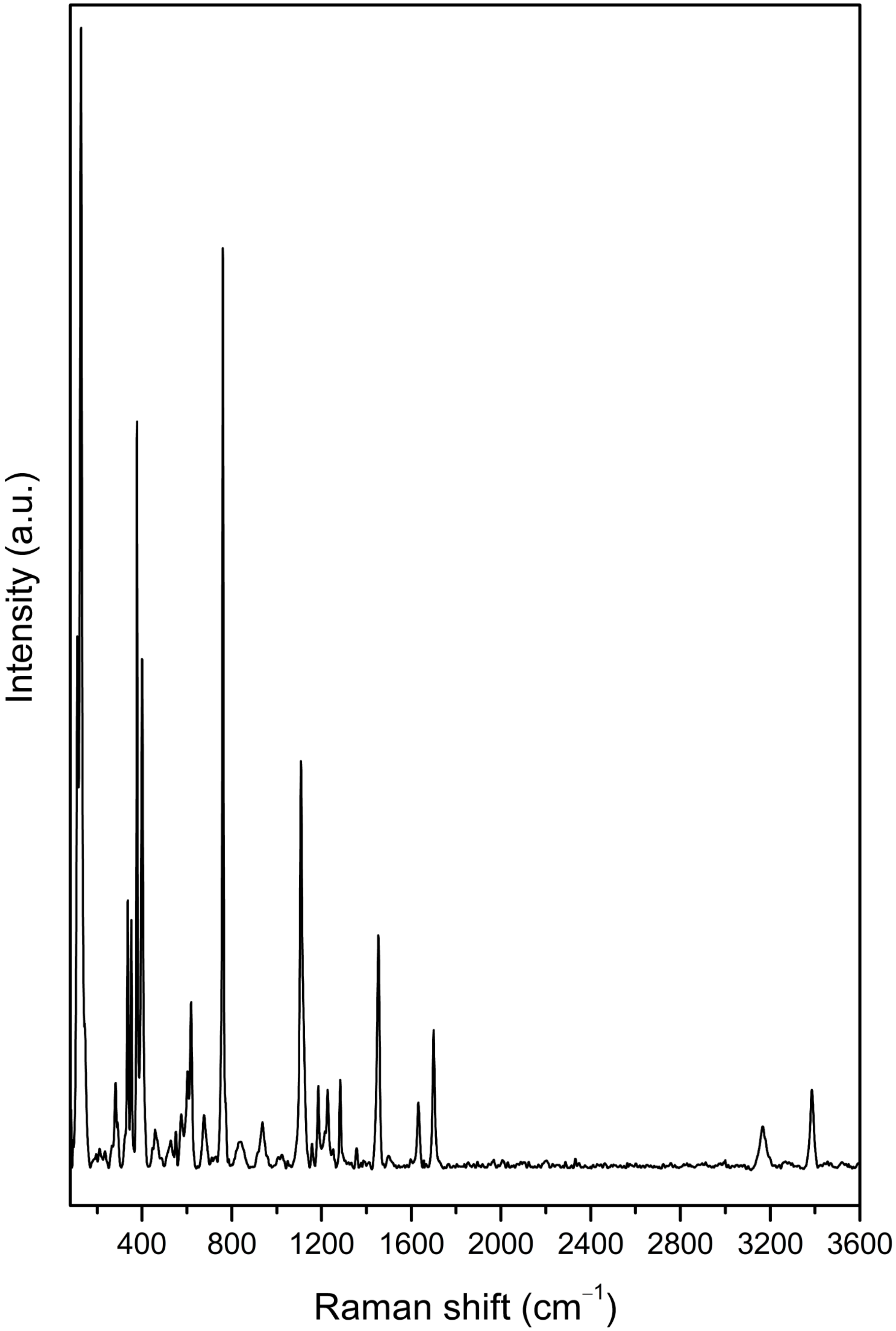


**Figure S35**. Raman spectrum of $C_3F_7CONH_2$ (−90 °C).

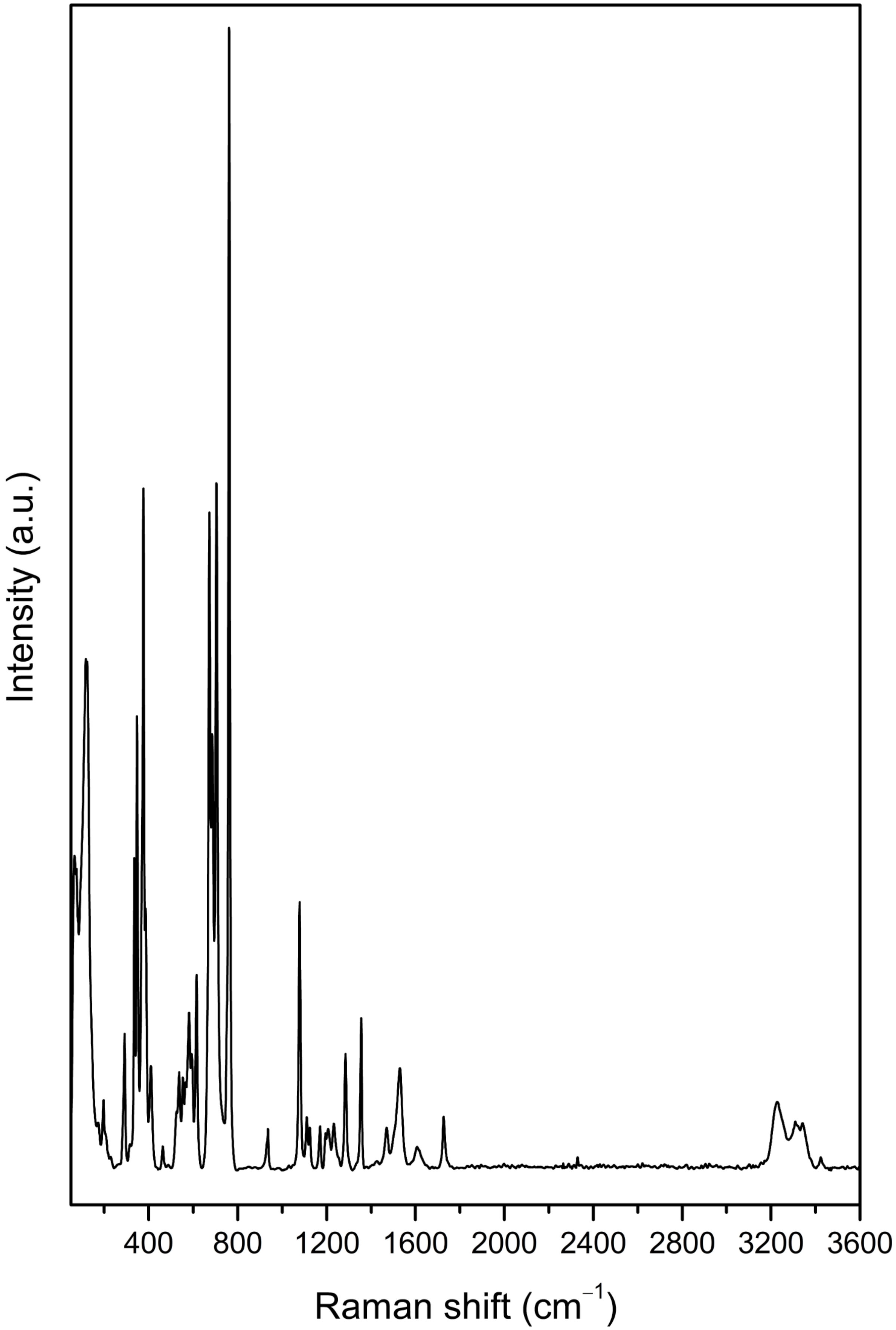


**Figure S36**. Raman spectrum of $C_3F_7C(OH)NH_2[AsF_6]$ (−100 °C).

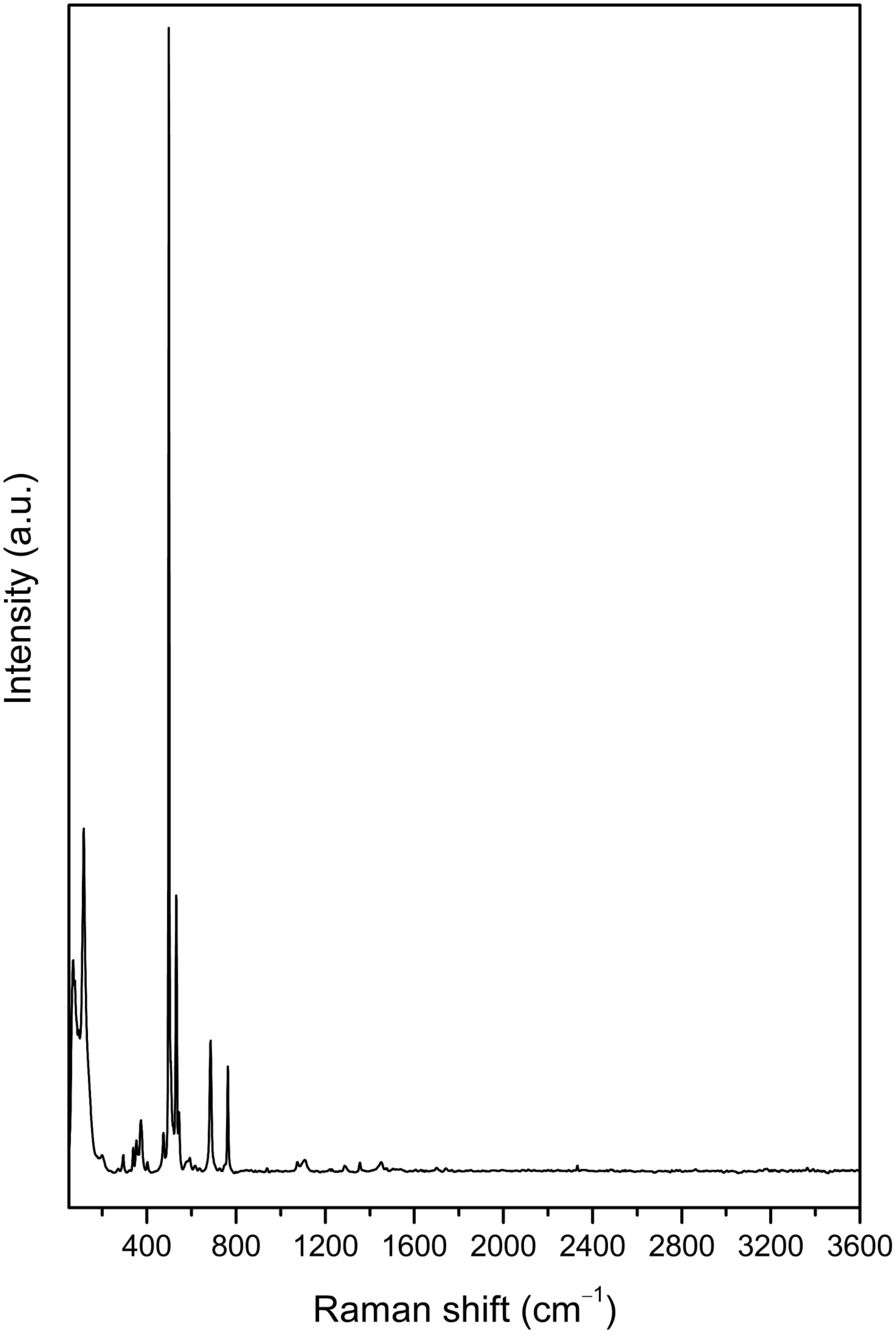


**Figure S37**. Raman spectrum of a mixture of $(C_3F_7CONH_2)_2H[AsF_6]$ and $C_3F_7C(OH)NH_2[AsF_6]\cdot XeF_2$ (−93 °C).

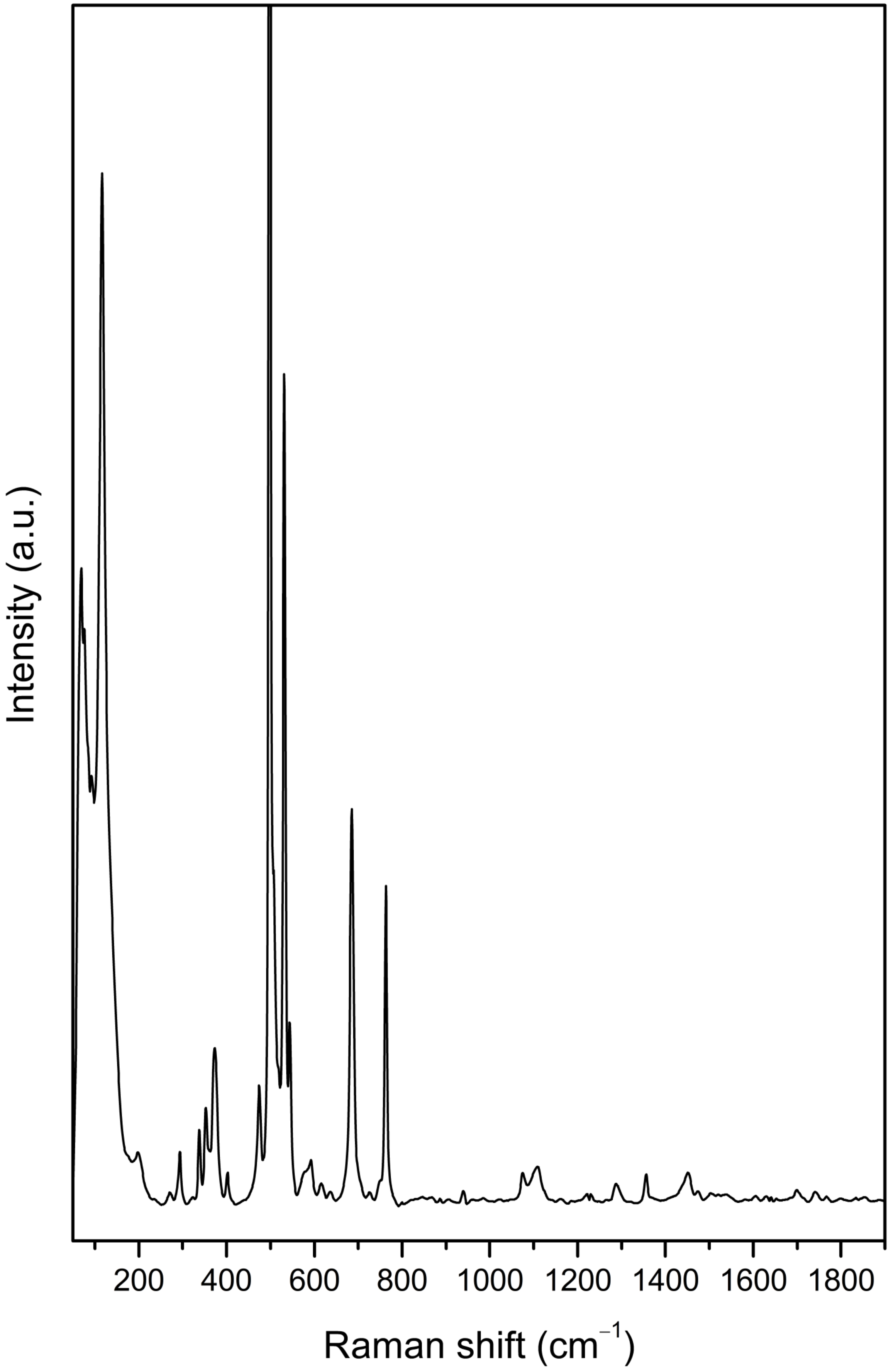


Figure S38. Enlarged Raman spectrum from Figure S37.

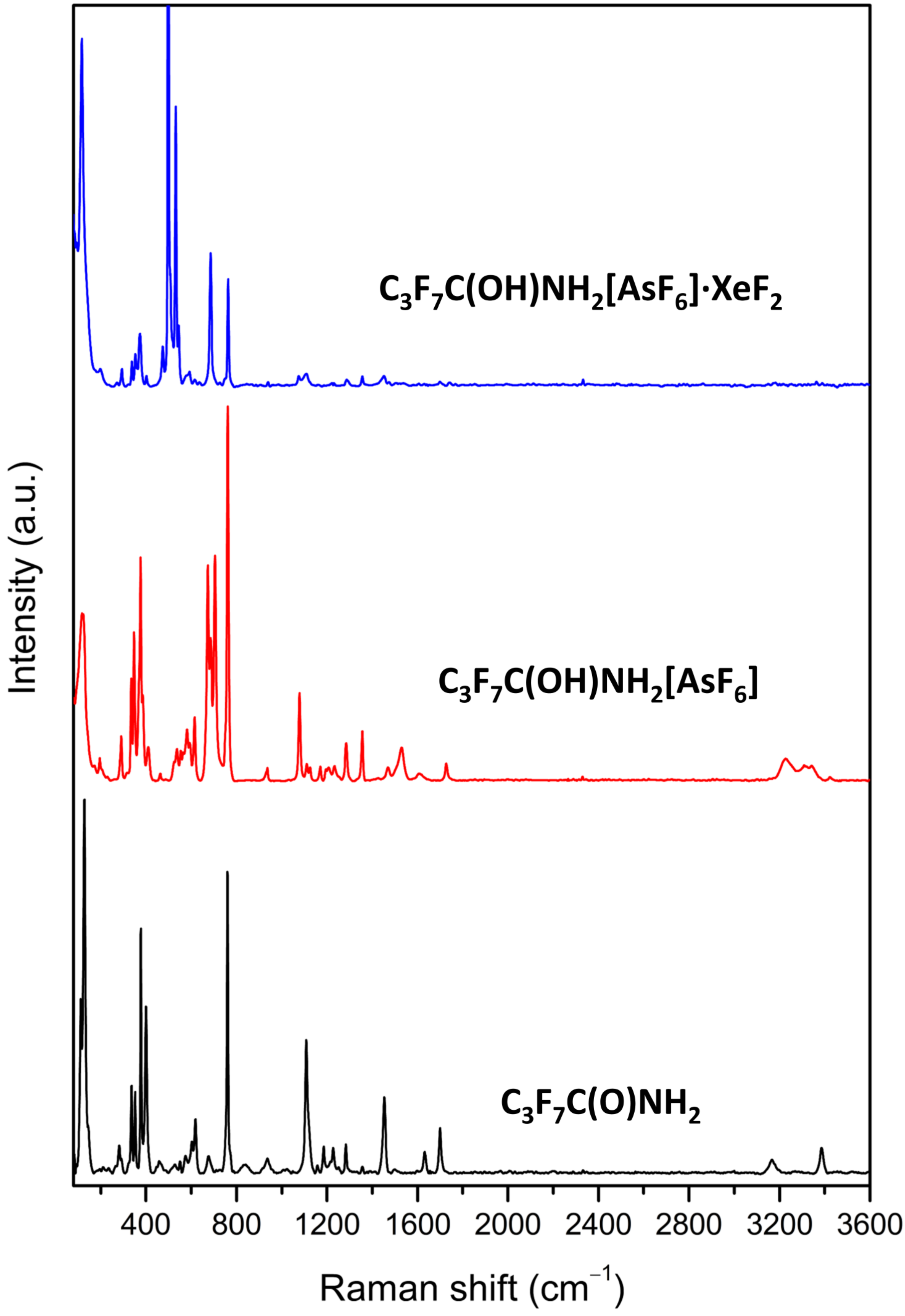


**Figure S39**. Comparison of Raman spectra of $C_3F_7CONH_2$, $C_3F_7C(OH)NH_2[AsF_6]$, and a mixture of $C_3F_7C(OH)NH_2[AsF_6]{\cdot}XeF_2$ and $(C_3F_7CONH_2)_2H[AsF_6]$.

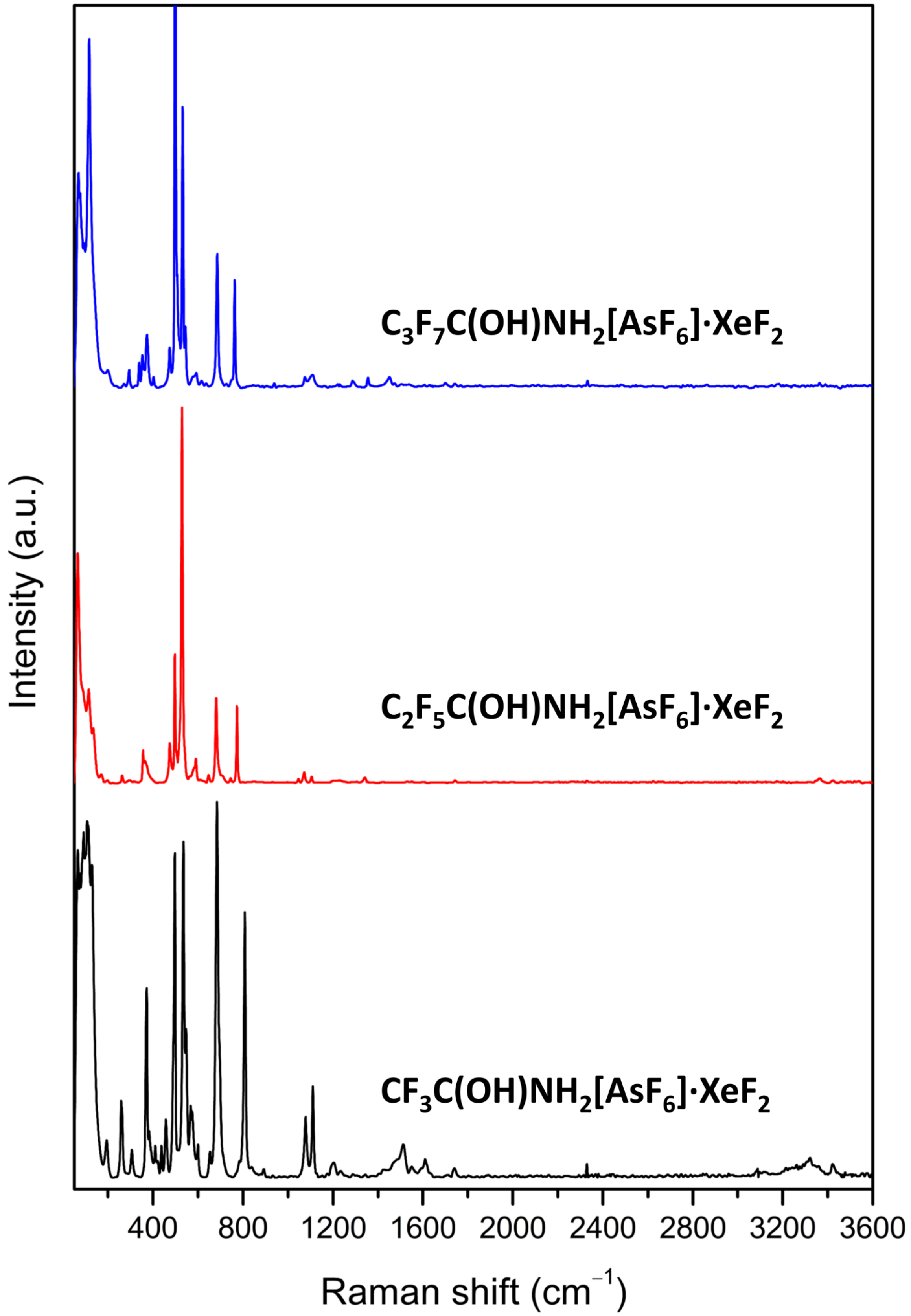


**Figure S40**. Comparison of Raman spectra (−90 °C) of $CF_3C(OH)NH_2[AsF_6]\cdot XeF_2$, $C_2F_5C(OH)NH_2[AsF_6]\cdot XeF_2$, and $C_3F_7C(OH)NH_2[AsF_6]\cdot XeF_2$. $(C_3F_7CONH_2)_2H[AsF_6]$ compound is also present in the Raman spectrum of $C_3F_7C(OH)NH_2[AsF_6]\cdot XeF_2$. Small peak at 2327 $cm^{-1}$ originates from the nitrogen stream.

# 3. Experimental details

***Caution:*** *All reagents ($XeF_2$, [XeF][$AsF_6$], anhydrous HF, $K_2NiF_6$, $AsF_5$, $F_2$) are toxic and highly corrosive and should be handled with extreme caution by trained personnel wearing appropriate protective clothing. Suitable first-aid equipment must be available at all times.*[2–4]

## Apparatus

All manipulations involving solid reagents were performed in a nitrogen-filled glovebox (Vigor SG1200/750E–SG1500/750E), whereas syntheses involving volatile materials were carried out on a fluorine-resistant metal vacuum line equipped with FEP (copolymer of tetrafluoroethylene and hexafluoroethylene) manifolds, a Monel Helicoid pressure gauge, a soda-lime scrubber, liquid-nitrogen-cooled traps, and a two-stage rotary vane pump. All reaction vessels were made of FEP, while the valves were made of either polychlorotrifluoroethylene (Kel-F) with an aluminium casing or polytetrafluoroethylene (PTFE) encased in brass. All vessels were passivated with $F_2$ overnight prior to use.

## Starting Materials

Fluorine gas (Solvay Fluor, 98–99%) was used without further purification. Commercial anhydrous HF (aHF, Linde, 99.995%) was distilled into a FEP vessel (outer diameter (o.d.) 19 mm, inner diameter (i.d.) 16 mm) and stored over $K_2NiF_6$ (Advanced Research Chemicals, 99.9%) prior to use. Trifluoroacetamide (Fluorochem, 99.0%), pentafluoropropionamide (Apollo Scientific, 97%) and heptafluorobutyramide (Fluorochem, 97.0%) were used as received. $XeF_2$ was synthesized photochemically from Xe and $F_2$ according to the literature procedure.[5] $AsF_5$ was synthesized by high-temperature fluorination of $As_2O_3$ and stored in a nickel vessel equipped with a nickel valve.[6] [XeF][$AsF_6$] was synthesized from $XeF_2$ and $AsF_5$ in aHF, following a procedure similar to that previously published.[7] Typically, $XeF_2$ (0.5 g, 2.95 mmol) was dissolved in aHF (1.6 mL) and $AsF_5$ (0.7 g, 4.43 mmol) was added at −196 °C. After thorough mixing at room temperature overnight, the excess aHF and remaining $AsF_5$ were removed at −65 ± 5 °C. The purity of the product was checked by powder X-ray diffraction and Raman spectroscopy.

## CHARACTERISATION

### Vibrational spectroscopy

Raman spectra were measured using a Horiba Jobin Yvon Labram-HR spectrometer coupled with an Olympus BXFM-ILHS microscope, employing either red or green lasers (633 or 532 nm, respectively). The typical spectral resolution was 4 $cm^{-1}$. The Raman spectra of all compounds were recorded directly on the aluminium trough used to mount the crystals for X-ray diffraction. The background subtraction was performed in the Bruker *OPUS* 8.7. software suite using concave rubberband correction (15 iterations, 50 baseline points).

### Single crystal X-ray diffraction (SCXRD)

Crystals were isolated directly from the FEP reaction vessels employing the following procedure. After the complete removal of solvent, the vessel was backfilled with nitrogen (> 1 bar) at approximately −78 °C and then detached from the vacuum system. It was subsequently cooled to −196 °C and tapped with a plastic stick to dislodge the crystals from the vessel walls. The vessel was then cut open, allowing the crystals to fall onto the aluminium trough of the low-temperature crystal-mounting apparatus, which was cooled by a stream of cold nitrogen (−50 to −100 °C).[8–10] Suitable crystals were selected under a microscope and were mounted on the tip of a MiTeGen loop using Fomblin oil (Z25, SynQuest).[11] The loop assembly was picked up with cryo-pin

tongs cooled to −196 °C and quickly transferred to the goniometer head, where the crystal was protected by the stream of cold nitrogen at 100 K.

Crystals of pentafluoropropionamide and heptafluorobutyramide suitable for SCXRD were obtained from the commercial reagents. A small amount of amide, which was stored in the glovebox, was transferred into a short piece of FEP tube (o.d. 6.0 mm; i.d. 4.7 mm) closed by FEP stopper/cap (o.d. 8.0 mm; i.d. 6.0 mm), and was then deposited onto the aluminium trough.

All crystals and powder were measured on a Rigaku OD XtaLAB Synergy-S Dualflex diffractometer equipped with a Dectris Eiger2 R CdTe 1M detector, PhotonJet-S microfocus Cu and Ag X-ray sources, and an Oxford Cryosystems 800 Series Cryostream. All datasets were processed with empirical and numerical absorption corrections using *CrysAlisPro* software.[12] Crystal structures were solved by the charge flipping method using *olex2.solve*,[13] whereas $(CF_3CONH_2)_2H[AsF_6]$ was solved with *SHELXT*,[14] and subsequently refined using *SHELXL*[15] within *Olex2*.[16] Figures were drawn using the program *DIAMOND*.[17]

Crystal data, data collection details, and structure refinement parameters for the structures determined by SCXRD are summarized in Table S1.

### Powder X-ray diffraction (PXRD)

The powder diffractogram of $CF_3C(OH)NH_2[AsF_6]\cdot XeF_2$ was measured at 100 K by mounting large amount of crystallites on the tip of a MiTeGen loop using Fomblin oil (Z25, SynQuest). Gandolfi movements were employed to enhance the random orientation of the crystallites. A measurement consisted of six frames, each with an exposure time of 300 s, with the detector positioned 90 mm away from the sample.

Calculated powder diffractogram of $CF_3C(OH)NH_2[AsF_6]\cdot XeF_2$ was obtained using *Mercury* 2025.2.0 (0.56087 Å, 2θ step = 0.01°, fwhm = 0.1).[18]

## SYNTHESES

### Synthesis of $CF_3C(OH)NH_2[AsF_6]$

An FEP vessel (4.7 mm i.d., 6 mm o.d.) was loaded with $CF_3CONH_2$ (76 mg, 0.67 mmol), and aHF (0.2 mL) was added at −196 °C. Upon warming to room temperature, a clear, colourless solution formed. The addition of Lewis acid $AsF_5$ (0.94 mmol) at −196 °C followed by warming to −45 °C yielded a clear solution. The solvent was removed under dynamic vacuum over the temperature range of −48 °C to −40 °C for one hour, affording a white, crystalline solid. The vessel was then backfilled with 900 Torr of $N_2$ at −78 °C and stored in dry ice prior to measurements. The material was subsequently transferred to an aluminium trough pre-cooled to −100 °C. The sample consisted of a white, chunky material, identified as $CF_3C(OH)NH_2[AsF_6]$.

### Synthesis of $CF_3C(OH)NH_2[AsF_6]\cdot XeF_2$

An FEP vessel (4.7 mm i.d., 6 mm o.d.) was loaded with $CF_3CONH_2$ (24 mg, 0.21 mmol), followed by the addition of $[XeF][AsF_6]$ (61 mg, 0.18 mmol) at ca. −120 °C. The vessel was transferred in liquid nitrogen to the vacuum system, and aHF (0.2 mL) was added. Upon warming to −60 °C, the white solid dissolved, yielding a clear, colourless solution. Slow solvent removal from −65 °C to −50 °C over nearly five hours afforded colourless crystals. The vessel was backfilled with 900 Torr of $N_2$ at −70 °C, and the material was transferred to an aluminium trough pre-cooled to −90 °C. The product consisted of white, chunky material, identified as the $CF_3C(OH)NH_2[AsF_6]\cdot XeF_2$ salt cocrystal. During crystal mounting, crystals of $H_3O[AsF_6]\cdot 2CF_3CONH_2$ and $(CF_3CONH_2)_2H[AsF_6]$ were also discovered. Targeted synthesis of these phases was not attempted. The PXRD pattern measured at 100 K (Figure S41) shows that $CF_3C(OH)NH_2[AsF_6]\cdot XeF_2$ is obtained in nearly pure form.

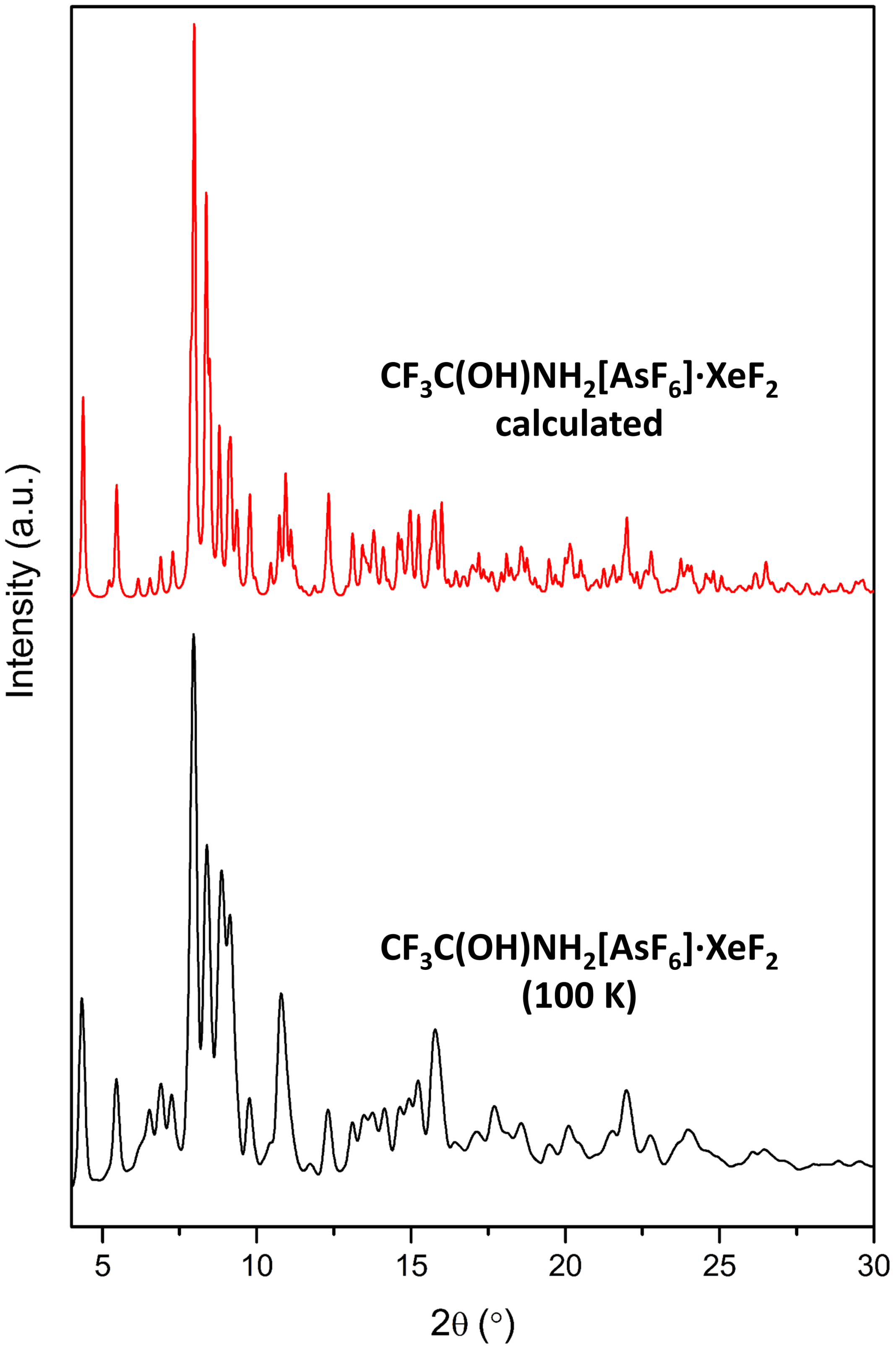


**Figure S41**. Experimental (black, measured at 100 K) and calculated (red) powder diffractogram of $CF_3C(OH)NH_2[AsF_6]·XeF_2$.

## Synthesis of $C_2F_5C(OH)NH_2[AsF_6]$

An FEP vessel (4.7 mm i.d., 6 mm o.d.) was loaded with $C_2F_5CONH_2$ (35 mg, 0.21 mmol), and aHF (0.3 mL) was added at −196 °C. Upon warming to room temperature a clear colourless solution formed. The addition of $AsF_5$ (0.25 mmol) at −196 °C, followed by warming to −30 °C, yielded a slightly turbid solution. Slow cooling to −78 °C afforded small, colourless needles. The solvent was removed under dynamic vacuum over the temperature range of −78 °C to −70 °C, affording a colourless, crystalline solid. The vessel was then backfilled with 900 Torr of $N_2$ at −70 °C, and the material was subsequently transferred to an aluminium trough pre-cooled to −50 °C. Although the material appeared dry inside the FEP vessel, residual HF was found adhering to the crystals. This layer evaporated after approximately one hour, leaving a considerable amount of powder on the crystals, which significantly diminished their quality.

## Synthesis of $C_2F_5C(OH)NH_2[AsF_6]_2{\cdot}XeF_2$

An FEP vessel (4.7 mm i.d., 6 mm o.d.) was loaded with $C_2F_5CONH_2$ (9 mg, 0.055 mmol), followed by the addition of $[XeF][AsF_6]$ (18 mg, 0.053 mmol) at ca. −120 °C. The vessel was transferred in liquid nitrogen to the vacuum system, and aHF (0.2 mL) was added. The vessel was then removed from the cooling bath and vigorously shaken for several seconds, yielding a clear, colourless solution. The vessel was backfilled with 1000 Torr of $N_2$ at −78 °C. Over a period of two weeks, colourless plates crystallized at −78 °C. Slow solvent removal between −78 °C to −70 °C afforded additional colourless crystals. The vessel was then backfilled with 1000 Torr of $N_2$ at −78 °C, and the material was transferred to an aluminium trough pre-cooled to −90 °C. Single crystals analysis has shown both $C_2F_5C(OH)NH_2[AsF_6]{\cdot}XeF_2$ and $XeF_2$, which was also confirmed with Raman spectroscopy.

The same compound can also be obtained by the reaction of the protonated amide and $XeF_2$. An FEP vessel (4.7 mm i.d., 6 mm o.d.) was loaded with $C_2F_5CONH_2$ (17 mg, 0.10 mmol), and aHF (0.5 mL) was added at −196 °C. $AsF_5$ (0.28 mmol) was added at −196 °C, and the mixture was then warmed to −50 °C, which yielded a white powder. The solvent was removed under dynamic vacuum over the temperature range of −50 °C to −30 °C. To the dry powder, aHF (0.2 mL) was added, followed by the addition of $XeF_2$ (19 mg, 0.11 mmol) by sublimation, producing a white, powdery material that was difficult to dissolve; only part of the sample dissolved at −25 °C. During slow cooling to −78 °C, a few block-like crystals formed. The residual solution was removed at −63 °C. SCXRD analysis of block-like crystals revealed them to be $C_2F_5C(OH)NH_2[AsF_6]_2{\cdot}XeF_2$. The Raman spectrum of the powder material was in good agreement with the Raman spectrum of the product obtained from the reaction of $C_2F_5CONH_2$ with $[XeF][AsF_6]$.

## Synthesis of $C_3F_7C(OH)NH_2[AsF_6]$:

An FEP vessel (4.7 mm i.d., 6 mm o.d.) was loaded with $C_3F_7CONH_2$ (39 mg, 0.18 mmol), and aHF (0.3 mL) was added at −196 °C. Upon warming to room temperature, a clear, colourless solution formed. $AsF_5$ (0.28 mmol) was added at −196 °C, and the mixture was then warmed to −25 °C, yielding a slightly turbid solution. Crystals formed at −78 °C after one hour. The solvent was removed under dynamic vacuum over the temperature range of −78 °C to −60 °C. The vessel was then backfilled with 1000 Torr of $N_2$ at −78 °C. The material was subsequently transferred to an aluminium trough pre-cooled to −100 °C. The sample consisted of a block-shaped crystals of $C_3F_7C(OH)NH_2[AsF_6]$. Even at 90 K, the anion remained disordered.

## Synthesis of $C_3F_7C(OH)NH_2[AsF_6]{\cdot}XeF_2$ and $(C_3F_7CONH_2)_2H[AsF_6]$:

An FEP vessel (4.7 mm i.d., 6 mm o.d.) was loaded with $C_3F_7CONH_2$ (19 mg, 0.09 mmol), followed by the addition of $[XeF][AsF_6]$ (27 mg, 0.08 mmol) at ca. −120 °C. The vessel was transferred in liquid nitrogen to the vacuum system, and aHF (0.2 mL) was added. Upon warming to −50 °C, the white solid dissolved, yielding a

clear, colourless solution. Slow cooling to −55 °C resulted in growth of large, colourless blocks, and further cooling to −65 °C afforded thin plates. The solvent was slowly removed under dynamic vacuum over the temperature range of −78 °C to −60 °C. The vessel was backfilled with 1000 Torr of $N_2$ at −78 °C, and the material was transferred to an aluminium trough pre-cooled to −93 °C. The product consisted of colourless, block-shaped crystals of $(C_3F_7CONH_2)_2H[AsF_6]$ and thin plates identified as the $C_3F_7C(OH)NH_2[AsF_6]{\cdot}XeF_2$ salt cocrystal. No attempts were made to isolate either component in phase-pure form.

## 4. References


[1] F. R. Fronczek and N. H. Fischer, CCDC 174252: Experimental Crystal Structure Determination, 2002, DOI: 10.5517/cc5vb1c.

[2] D. Peters and R. Miethchen, *J. Fluorine Chem.*, 1996, **79**, 161−165.

[3] E. B. Segal, *Chem. Health Saf.*, 2000, **7**, 18−23

[4] M. Möbs and F. Kraus, *ACS Chem. Health Saf.*, 2025, **32**, 662–672.

[5] A. Šmalc and K. Lutar, *Inorg. Synth.*, 1992, **29**, 1–4.

[6] Z. Mazej and B. Žemva, *J. Fluorine Chem.,* 2005, **126**, 1432–1434.

[7] R. J. Gillespie and B. Landa, *Inorg. Chem.,* 1973, **12**, 1383–1388.

[8] M. Veith and H. Bärnighausen, *Acta Crystallogr.,* 1974, B**30**, 1806–1813.

[9] M. Lozinšek, H. P. A. Mercier and G. J. Schrobilgen, *Angew. Chem. Int. Ed.,* 2021, **60**, 8149–8156.

[10] K. Motaln, K. Gurung, P. Brazda, A. Kokalj, K. Radan, M. Dragomir, B. Žemva, L. Palatinus and M. Lozinšek, *ACS Cent. Sci.,* 2024, **10**, 1733–1741.

[11] K. Motaln, E. Uran, N. Giordano, S. Parsons and M. Lozinšek, *J. Appl. Crystallogr.*, 2025, **58**, 221–226.

[12] Rigaku OD, CrysAlis PRO. Rigaku Corporation, Wrocław, Poland, 2025.

[13] L. J. Bourhis, O. V. Dolomanov, R. J. Gildea, J. A. K. Howard and H. Puschmann, *Acta Crystallogr.*, 2015, A**71**, 59–75.

[14] G. M. Sheldrick, *Acta Crystallogr.,* 2015, A**71**, 3–8.

[15] G. M. Sheldrick, *Acta Crystallogr.,* 2015, C**71**, 3–8.

[16] O. V. Dolomanov, L. J. Bourhis, R. J. Gildea, J. A. K. Howard and H. Puschmann, *J. Appl. Crystallogr.*, 2009, **42**, 339–341.

[17] K. Brandeburg, Diamond – Crystal and Molecular Structure Visualization, Crystal Impact GbR, Bonn, Germany, 2022.

[18] C. F. Macrae, I. Sovago, S. J. Cottrell, P. T. A. Galek, P. McCabe, E. Pidcock, M. Platings, G. P. Shields, J. S. Stevens, M. Towler and P. A. Wood, *J. Appl. Crystallogr.*, 2020, **53**, 226–235.
